# Sparstolonin B, a Novel Plant Derived Compound, Arrests Cell Cycle and Induces Apoptosis in N-Myc Amplified and N-Myc Nonamplified Neuroblastoma Cells

**DOI:** 10.1371/journal.pone.0096343

**Published:** 2014-05-01

**Authors:** Ambrish Kumar, Daping Fan, Donald J. DiPette, Ugra S. Singh

**Affiliations:** 1 Department of Pathology, Microbiology and Immunology, School of Medicine, University of South Carolina, Columbia, South Carolina, United States of America; 2 Department of Cell Biology and Anatomy, School of Medicine, University of South Carolina, Columbia, South Carolina, United States of America; 3 Department of Internal Medicine, School of Medicine, University of South Carolina, Columbia, South Carolina, United States of America; Rutgers - New Jersey Medical School, United States of America

## Abstract

Neuroblastoma is one of the most common solid tumors and accounts for ∼15% of all the cancer related deaths in the children. Despite the standard therapy for advanced disease including chemotherapy, surgery, and radiation, the mortality rate remains high for these patients. Hence, novel therapeutic agents are desperately needed. Here we examined the anticancer activity of a novel plant-derived compound, sparstolonin B (SsnB; 8,5′-dihydroxy-4-phenyl-5,2′-oxidoisocoumarin) using neuroblastoma cell lines of different genetics. SsnB was recently isolated from an aquatic Chinese herb, *Sparganium stoloniferum*, and tubers of this herb have been used in traditional Chinese medicine for the treatment of several inflammatory diseases and cancers. Our cell viability and morphological analysis indicated that SsnB at 10 µM concentration significantly inhibited the growth of both N-myc amplified (SK-N-BE(2), NGP, and IMR-32 cells) and N-myc nonamplified (SH-SY5Y and SKNF-1 cells) neuroblastoma cells. The flow cytometric analyses suggested that SsnB arrests the cell cycle progression at G2-M phase in all neuroblastoma cell lines tested. Exposure of SsnB inhibited the compact spheroid formation and reduced the tumorigenicity of SH-SY5Y cells and SK-N-BE(2) cells in *in vitro* 3-D cell culture assays (anchorage-independent colony formation assay and hanging drop assay). SsnB lowers the cellular level of glutathione (GSH), increases generation of reactive oxygen species and activates the cleavage of caspase-3 whereas co-incubation of a GSH precursor, N-acetylcysteine, along with SsnB attenuates the inhibitory effects of SsnB and increases the neuroblastoma cell viability. Our results for the first time demonstrate that SsnB possesses anticancer activity indicating that SsnB-induced reactive oxygen species generation promotes apoptotic cell death in neuroblastoma cells of different genetic background. Thus these data suggest that SsnB can be a promising drug candidate in neuroblastoma therapy.

## Introduction

Sparstolonin B (SsnB) is a novel plant derived active compound recently isolated from the tubers of an aquatic herb, *Sparganium stoloniferum* [1]. *In vivo* and *in vitro* studies revealed its anti-inflammatory [1], [2] and anti-angiogenic [3] properties. SsnB acts as an antagonist to Toll-like Receptors 2 and 4 (TLR2 and TLR4), and exhibits anti-inflammatory property by selectively inhibiting TLR2 and TLR4-triggered inflammatory response in mouse and human macrophages [1], [2]. In traditional Chinese medicine (TCM), the tubers of this herb have been used for the treatment of several inflammatory diseases, and the crude extract prepared form this herb has anti-spasmodic and anti-tumor properties [4]–[6]. As revealed by NMR and X-ray crystallography, SsnB (8,5′-dyhydroxy-4-phenyl-5,2′-oxidoisocoumarin) is a polyphenol with structural similarities to isocoumarins and xanthone. Isocoumarins and xanthone family of compounds are well known for their anti-inflammatory and anti-tumor properties [7]–[10]. Due to the structural similarities of SsnB with isocoumarins and xanthone, we decided to examine the anticancerous properties of SsnB.

Neuroblastoma is a malignant pediatric cancer of the postganglionic sympathetic nervous system and derived from the neural crest cells during embryonic development. Initially it develops in the adrenal gland and metastasizes to liver, bone, bone marrow, lymph nodes, neck and chest. It is the most common cancer in babies younger than one and second most common tumor in children [11], [12]. It accounts for 7% of all childhood cancers (Cancer Facts & Figures 2013. Atlanta, GA: American Cancer Society, 2013), and is responsible for 15% of all cancer deaths in children younger than 15 years. About 30%–50% of children with high-risk neuroblastoma experience long-term survival.

Neuroblastoma tumor comprises of various heterogeneous population of cells which differ at morphological, biochemical and genetic levels [13]–[15]. Genomic amplification of N-myc gene, rearrangement or deletion of distal region of the chromosome 1 (1p31-arm) [16], [17] or alterations in chromosomes 11, 14 and 17 [18], [19] are most common cytogenetic features identified in low to advance stages of neuroblastomas. Mutations in tumor suppresser genes, i.e., p53, retinoblastoma, RET, p16, p18 or p27 have been reported to promote tumorigenesis [20]–[22]. These karyotype and cytogenetic alterations render tumors resistant to available chemotherapies [23]. For example, retinoic acid induces neuronal differentiation in neuroblastoma cells [24], [25] and commonly used as in residual therapy. However, neuroblastoma cells with N-myc amplified oncogene do not respond to retinoic acid [26], [27]. Therefore, it is crucial to find new therapeutic agents that can exhibit anti-proliferative effects on neuroblastoma cells irrespective of their genetic abnormalities.

In the present study, for the first time we have reported the anticancer activity of SsnB and have demonstrated that SsnB inhibits the growth of human neuroblastoma cells of different genetic background by arresting cell cycle progression and by inducing apoptotic cell death through generation of reactive oxygen species.

## Materials and Methods

### Human Neuroblastoma Cell Culture and SsnB Treatments

Human neuroblastoma cell lines (SH-SY5Y, IMR-32, SK-N-BE(2) and SKNF-1 cells) were obtained from The American Type Culture Collection (ATCC; Manassas, VA), and NGP cells were kind gift from Garrett M. Brodeur (The Children’s Hospital of Philadelphia, Philadelphia, Pennsylvania) [28]. All cell lines were maintained in complete Dulbecco’s Modified Eagle’s Medium (DMEM) supplemented with 10% fetal bovine serum (FBS; Atlanta Biologicals, Lawrenceville, GA) and 1× antibiotic-antimycotic solution (containing 100 U/ml penicillin, 100 µg/ml streptomycin and 0.25 µg/ml amphotericin B), and grown at 37°C in a humidified incubator with 5% CO_2_. Stock solution of SsnB was prepared in dimethyl sulfoxide (DMSO). Cells treated with different concentrations of SsnB in DMEM with 10% FBS were grown for subsequent days. Cells treated with equal volume of DMSO were used as control.

### Cell Viability Assay

The viability of SsnB-treated cells was determined by MTT assay following manufacturer’s instructions (Roche diagnostics corporation, Indianapolis, IN). Briefly, cells (1×10^4^ cells/well) grown in 96-well cell culture plate were incubated with SsnB in 100 µl of complete culture medium with 10% FBS. After treatments, cells were incubated with 10 µl of MTT reagent for 4 h and then incubated overnight in solubilization buffer (100 µl) at 37°C. Absorbance of the formazan product was read at 575 nm in spectramax spectrophotometer (Molecular Devices, Sunnyvale, CA). A reference wavelength of 690 nm was used to detect background. The measured absorption directly correlates with the number of viable cells in culture. The experiments were performed in triplicates and repeated at least three times.

### Cell Cycle Analyses by Flow Cytometry

The cell cycle progression was determined by flow cytometer analysis [29]. Neuroblastoma cells were treated with SsnB or DMSO in DMEM with 10% FBS. Cells were collected after trypsinization with trypsin-EDTA solution (Atlanta Biologicals) and washed with 1× phosphate buffered saline (PBS). Approximately 1×10^6^ cells were suspended in 300 µl of ice-cold 1× PBS, fixed by adding 700 µl of chilled absolute ethanol (to make final ethanol concentration 70%) drop wise and incubated for overnight at −20°C. Cells were pelleted at 1000× rpm for 5 min at 4°C, washed with 1× PBS for 3 times and stained with propidium iodide (Sigma-Aldrich, St. Louis, MO) by adding 1 ml of propidium iodide staining solution (containing 20 µg/ml propidium iodide and 10 µg/ml DNase-free RNase A in 0.1% Triton X-100/PBS) for 1 h on ice in dark. Samples were subjected to fluorescence-activated cell sorting analyses utilizing Beckman Coulter flow cytometer (Beckman Coulter, Indianapolis, IN). A minimum of 10,000 events were analyzed in each experiment.

### Immunofluorescence Microscopy

For immunofluorescence staining, cells grown in Lab-TekII chamber slides (Fisher Scientific, Pittsburgh, PA) were treated with SsnB compound in complete culture medium. After treatments, cells were washed with 1× PBS, fixed with 4% paraformaldehyde/PBS for 20 min, and permeabilized with 0.2% Triton X-100/PBS for 10 min at room temperature. Cells were washed with 1× PBS for 3 times and blocked with 5% immunoglobulin (IgG) free-bovine serum albumin (BSA; Jackson ImmunoResearch Laboratories, West Grove, PA) in 1× PBS for overnight at 4°C. Cells were incubated with antibody raised against active form of caspase-3 (Cell Signaling Technology, Danvers, MA) diluted in 2.5% BSA/PBS for overnight at 4°C. Primary antibodies were detected with secondary antibodies conjugated with fluorescein isothiocyanate (FITC; Santa Cruz Biotechnology, Dallas, TX) for 2 h at room temperature. After washing with 1× PBS, cells were mounted with antifade Vectashield mounting media (Vector Laboratories, Burlingame, CA), and signals were visualized under Nikon-E600 fluorescence microscope. Nuclei were counterstained with 4′,6-diamidino-2-phenylindole (DAPI; Sigma).

### Total Cell Protein Isolation and Western Blot Analyses

After treatment with SsnB, cells were collected, washed with 1× PBS, and lysed with 1× cell lysis buffer (Cell Signaling Technology, Danvers, MA) containing phenylmethylsulfonyl fluoride (PMSF) and protease inhibitors (aprotinin and leupeptin) for 30 min on ice, and centrifuged at 12000× rpm for 10 min at 4°C. Supernatant (total cell protein) was collected and stored at −80°C. Protein concentration was determined by bicinchoninic acid method using BCA protein assay kit (Pierce/ThermoScientific, Waltham, MA). Equal amount of proteins were diluted with 5 x Laemmli samples loading buffer and boiled for five minutes. The proteins were subjected to sodium dodecyl sulfate (SDS)-polyacrylamide gel electrophoresis and analyzed by Western blots [30]. Briefly, after electrophoresis, proteins were transferred on polyvinylidene difluoride (PVDF) membrane at 100 volt for 3 h in cold room. Membrane was blocked with 5% non-fat dry milk/TBST (20 mM Tris-Cl, pH 7.4; 150 mM NaCl with 0.1% Tween-20) for 4 h at room temperature followed by incubation in primary antibodies diluted in 2.5% non-fat dry milk/TBST for overnight at 4°C. After washing with TBST, membrane was incubated with secondary antibodies (horseradish peroxidase-conjugated goat anti-rabbit or goat anti-mouse IgG; Santa Cruz Biotechnology, Dallas, TX) diluted in 2.5% non-fat dry milk/TBST for 3 h at room temperature. Signals were detected by chemiluminiscence detection kit (Pierce/Thermo Fisher Scientific, Rockford, IL). Primary antibodies used were cleaved caspase-3 and β-actin (Cell Signaling Technology), N-myc and p53 (Santa Cruz Biotechnology).

### Detection of Reactive Oxygen Species Generation

The production of reactive oxygen species (ROS) in SsnB-treated neuroblastoma cells was detected by a cell-permeable fluorescence probe H_2_DCFDA (carboxy-2′,7′-dichlorodihydrofluorescein diacetate) according to the manufacturer’s instructions (Invitrogen, Carlsbad, CA) [31]. Briefly, neuroblastoma cells were cultured in DMEM with 10% FBS in Lab-TekII chamber slides (Fisher Scientific, Pittsburg, PA) and treated with SsnB for 2 days (SH-SY5Y and IMR-32 cells), 3 days (NGP cell) or 4 days (SKNF-1 and SK-N-BE(2) cells). After treatments, cells were washed with Hank’s buffered salt solution (HBSS; Thermo Scientific, Waltham, MA) and incubated with 25 µM of carboxy-H_2_DCFDA dye for 30 min at 37°C in dark. Nuclei were counterstained with DAPI. Cells were washed three times with HBSS, mounted with Vectashield mounting medium (Vector Laboratories), and immediately examined under Nikon-E600 fluorescence microscopy equipped with fluorescein isothiocyanate (FITC) filter [32]. Oxidation of H_2_DCFDA probe occurs almost exclusively in the cytosol, and generates a fluorescence that is proportional to ROS generation in that cell. As a negative control, cells without H_2_DCFDA dye were used to detect autofluorescence. The fluorometric analysis to detect generation of ROS was performed as described previously [33]. Briefly, after treatments with SsnB as mentioned above, cells were labelled with carboxy-H_2_DCFDA dye (25 µM) for 30 min at 37°C. Cells were trypsinized, washed, and re-suspended in PBS and fluorescence intensity was monitored at excitation wavelength 485 nm and emission wavelength 530 nm with a spectramax spectrophotometer (Molecular Devices, Sunnyvale, CA).

### Colony Formation Assay

A two layer soft agarose assay system was used in these studies with some modifications [34]. Approximately 1×10^4^ cells were suspended in 0.35% agarose in DMEM with 10% FBS, and were gently laid on the bottom agarose layer containing 0.8% agarose with DMEM+10% FBS in 35 mm^2^ cell culture dishes. Once solidified, the dishes were incubated at 37°C in a humidified chamber with 5% CO_2_. Next day, 500 µl of complete cell culture medium with respective concentrations of SsnB was added, and cells were allowed to grow for 45 days in an incubator chamber. Fresh medium with respective concentrations of SsnB was added to the dishes in every 5 days. Cells treated with equal volume of DMSO were used as control. At the end of experiments, cells were stained with 0.1% crystal violet and cell colonies (more than 15 cells per colony) were counted under the light microscope.

### Hanging-drop Assay for Spheroid Formation

3-D *in vitro* hanging drop assay was carried out to detect the SsnB effect in neuroblastoma spheroid formation [35]. Neuroblastoma cells were prepared as single cell suspension in complete culture medium (DMEM+10% FBS) without or with SsnB (1 and 10 µM). Twenty microliter drop of each prepared cell suspension containing 20,000 cells/drop were pipetted into the inner side of the lid of a 60 mm diameter tissue culture dish. The lid was gently inverted and placed on top of the culture dish filled with 5 ml of sterile 1× PBS to humidify the culture chamber. The drops were incubated under tissue culture conditions (at 37°C and 5% CO_2_) allowing the cells to form aggregate at the base of the droplet. The cells in each droplet were photographed using Olympus SZX2 stereo microscope (Olympus America Inc., Center Valley, PA).

### Measurement of Glutathione (GSH) Level

Cells treated with SsnB or DMSO were washed and re-suspended in PBS and the intracellular GSH level was measured by luminescence based GSH-Glo glutathione assay kit (Promega, Madison, WI) following manufacturer’s protocol. The luminescence was monitored in a luminometer (Promega Biosystems, Sunnyvale, CA).

### Data Analysis

Data are presented as the mean and standard deviation (S.D.) of at least three independent experiments. Comparisons were made among the groups using one-way ANOVA followed by Tukey-Kramer ad hoc test (GraphPad software, La Jolla, CA). A *p*-value<0.05 was considered significant.

## Results

### SsnB Inhibits the Survival of Neuroblastoma Cells

**Figure 1:**
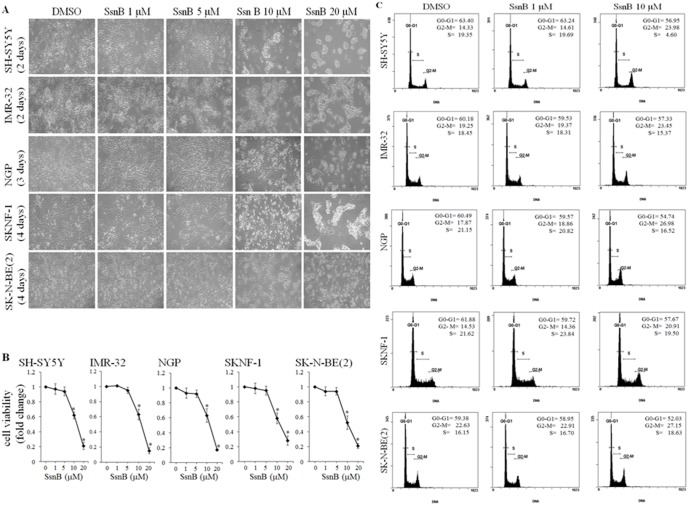
SsnB inhibits cell growth and viability of neuroblastoma cells. (A) Phase contrast images showing the morphology of neuroblastoma cells after treatments with SsnB. Neuroblastoma cells (SH-SY5Y, IMR-32, NGP, SKNF-1 and SK-N-BE(2) cells) were grown in presence of DMSO or SsnB (1, 5, 10, and 20 µM) in complete culture medium and were photographed on indicated times. (**B**) Bar diagrams showing the cell viability after SsnB treatment as evaluated by MTT assays. Neuroblastoma cells were treated with SsnB (1, 5, 10, and 20 µM) for 2 days (SH-SY5Y and IMR-32), 3 days (NGP) or 4 days (SKNF-1 and SK-N-BE(2) cells) and cell viability was measured by MTT assay at 575 nm. Data are represented in fold change and *p<0.05 *vs* control. (**C**) SsnB arrest cell cycle at G2/M phase. Representative histograms illustrating the cell cycle progression of neuroblastoma cells in presence of SsnB. Neuroblastoma cells treated with SsnB (1 µM or 10 µM) in DMEM with 10% FBS for 2 days (SH-SY5Y and IMR-32), 3 days (NGP) or 4 days (SKNF-1 and SK-N-BE(2) cells) were labelled with propidium iodide and cell cycle stage was analyzed by flow cytometry.

To test the inhibitory effects of SsnB on neuroblastoma cell survival, we treated human neuroblastoma cell lines of different genetic background (SH-SY5Y, SKNF-1, NGP, IMR-32 and SK-N-BE(2) cells) with 1 µM, 5 µM, 10 µM or 20 µM concentration of SsnB under *in vitro* conditions. Following SsnB treatments, neuroblastoma cell were grown for subsequent days and were photographed under light microscope to evaluate the cell morphology. The phase contrast images as shown in Figure 1A demonstrated that SsnB alters the cellular appearance and promotes cell death (as evaluated by cell morphology; round and clumping of cells is indication of cell death) in all these neuroblastoma cell lines. SsnB at 10 µM and above concentrations changed the morphology (as evaluated by rounded cells and indication of cell death) of SH-SY5Y cells and IMR-32 cells (after 2 days treatment), NGP (after 3 days treatment), SKNF-1 and SK-N-BE(2) cells (after 4 days treatment) compared to control DMSO-treated cells. However, such morphological changes were not significant at 1 or 5 µM conc. of SsnB in any cell line tested.

We further carried out MTT assay to examine the effects of SsnB on viability of all the above listed neuroblastoma cells. After 2 days (SH-SY5Y and IMR-32), 3 days (NGP cells) or 4 days (SKNF-1 and SK-N-BE(2) cells) treatment with SsnB (1, 5, 10 and 20 µM), MTT cell viability assays were performed. The bar diagram in Figure 1B showed that SsnB inhibited, effectively and dose-dependently, the viability of all neuroblastoma cell lines tested. Compared to DMSO, SsnB at and above 10 µM concentration significantly reduced the viability of these cell lines (*p<0.05 *vs* DMSO) and the IC_20_ and IC_50_ were found to be in the range of 6–8 µM and 10–12 µM, respectively. We did not observe significant difference in cell survival at SsnB at 1 µM or 5 µM concentrations compared to control DMSO-treated neuroblastoma cells (*p*>0.05 *vs* DMSO). No cytotoxic response of SsnB at these concentrations (and up to ∼100 µM) was observed in various normal human cells e.g. human monocytic THP-1 cells, phorbol 12-myristate 13-acetate-differentiated THP-1 macrophages, human umbilical vein endothelial cells and human aortic smooth muscle cells as reported in reference [1]. These data demonstrate the anti-proliferative activity of SsnB on human neuroblastoma cells, and suggest that SH-SY5Y and IMR-32 cells were most sensitive, NGP cell was moderately sensitive, SKNF-1 and SK-N-BE(2) cell lines were least sensitive towards SsnB treatment. Since 50% cell viability was observed in range of 10–12 µM SsnB conc., we used 10 µM as higher conc. and 1 µM lower conc. in our further experiments.

### SsnB Regulates Cell Cycle Progression in Neuroblastoma Cells

To test whether SsnB-induced cell growth inhibition occurs through cell cycle arrest, we analyzed cell cycle distribution by flow cytometry after DNA staining with propidium iodide. Representative histogram in Figure 1C showed that 2 days (SH-SY5Y, IMR-32) or 3 days (NGP cells) exposure of SsnB (10 µM) resulted in increase of G2-M phase cells when compared with DMSO-treated controls (G2/M for SH-SY5Y, SsnB 10 µM = 23.98% *vs* control = 14.33%; for IMR-32, SsnB 10 µM = 23.45% *vs* control = 19.25%; and for NGP, SsnB 10 µM = 26.98% *vs* control = 17.87%). Similarly, SsnB treatment for 4 days increases the number of SK-N-BE(2) cells and SKNF-1 cells in G2/M phase, compared to control (G2/M for SKNF-1, SsnB 10 µM  = 14.53% *vs* control = 20.91%; for SK-N-BE(2) cells, SsnB 10 µM = 27.15% *vs* control = 22.26%). The SsnB (10 µM) induced increase in G2/M fraction was accompanied by a decrease in G0/G1 phase cells. SsnB at 1 µM concentration did not affect cell cycle progression compared to DMSO-treated controls. These results indicated that the exhibited anti-proliferative effect of SsnB (10 µM) may be exerted by cell cycle arrest at G2-M transition in these neuroblastoma cell lines.

### SsnB Generates Reactive Oxygen Species (ROS) in Neuroblastoma Cells

**Figure 2:**
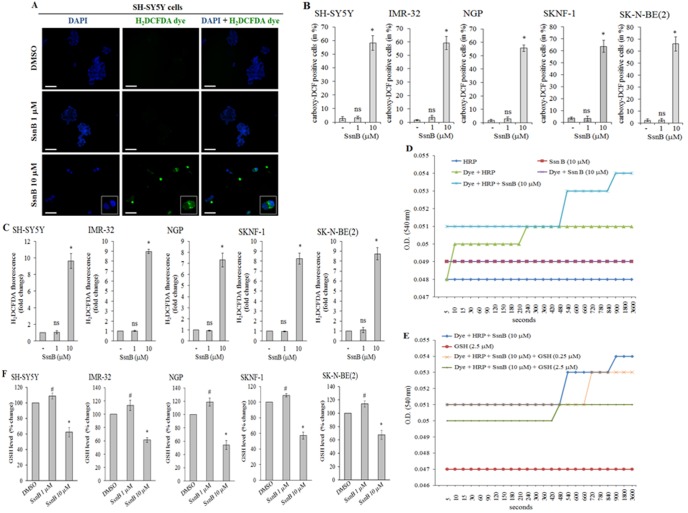
SsnB induces reactive oxygen species (ROS) generation. ROS levels were evaluated by H_2_DCFDA staining after SsnB treatments (1 µM or 10 µM) for 2 days (SH-SY5Y, IMR-32), 3 days (NGP) or 4 days (SKNF-1 and SK-N-BE(2) cells). Signals were examined under fluorescence microscope (**A**); carboxy-DCF positive cells (green) were quantitated by ImageJ programme and plotted (**B**). (**A**) Representative fluorescence images from SH-SY5Y cells showing that compared to DMSO and SsnB (1 µM), SsnB at 10 µM concentration induced ROS generation (green, H_2_DCFDA-positive cells) and signals were exclusively present in cytoplasm (as shown in enlarged images). Nuclei were counterstained with DAPI (blue). Images are representative of at least three independent experiments. Scale bar = 100 µm. (**C**) Bar diagrams representing the fold change in fluorescence intensity of H_2_DCFDA in cells treated with or without SsnB (1 µM or 10 µM) as mentioned above. After labelling with H_2_DCFDA dye (25 µM) for 30 min fluorescence intensity was monitored at excitation wavelength 485 nm and emission wavelength 530 nm. Values are mean and S.D. of three experiments. *p<0.05, SsnB 10 µM *vs* control; ns  =  nonsignificant *vs* control. (**D** and **E**) ROS generating capacity of SsnB was also evaluated in cell-free system containing H_2_DCFDA (25 µM), horseradish peroxidase (HRP, 5 units/ml), SsnB (10 µM) and GSH (0.25 or 2.5 µM) alone or in combinations. Optical density (OD) at 540 nm were taken at different time points and plotted. (**F**) Neuroblastoma cells treated with SsnB for 2, 3 or 4 days as mentioned above were used to measure cellular glutathione (GSH) levels using luminescence based glutathione assay kit. Values were expressed in % change compared to control samples (considering control as 100%) and plotted. Bar represents mean and S.D. of three independent experiments and *p<0.05, SsnB 10 µM *vs* control; #*p*≤0.05, SsnB 1 µM *vs* control.

Isocoumarin and coumarin derivative compounds are well known to exhibit their anti-tumor effects by reactive oxygen species-induced cell death [36], [37]. To test whether SsnB-induced cell death is resulted from increased level of reactive oxygen species (ROS), we performed H_2_DCFDA staining [31], [32]. H_2_DCFDA is a cell-permeable non-fluorescent indicator for ROS that is oxidized in presence of reactive oxygen species to fluorescent molecule carboxy-DCF. Neuroblastoma cells treated with SsnB for 2 days (SH-SY5Y and IMR-32), 3 days (NGP cells) or 4 days (SKNF-1 and SK-N-BE(2) cells) were labelled with H_2_DCFDA dye for 30 min and cells were immediately examined under fluorescence microscopy. The carboxy-DCF positive cells were quantitated by ImageJ software and plotted. The representative fluorescence images of SH-SY5Y cells in Figure 2A indicated that the number of carboxy-DCF positive cells (green) was significantly increased at 10 µM concentration of SsnB compared to DMSO-treated samples (*p<0.05 *vs* DMSO; Figure 2B). The ROS generation was observed exclusively in those SsnB treated cells which contain fragmented nuclei (blue). Similar to SH-SY5Y cells, SsnB (10 µM) significantly increased carboxy-DCF positive cells in other neuroblastoma cell lines tested (bar diagram in Figure 2B; *p<0.05 *vs* DMSO). In negative control, no such green staining was observed in cells processed without H_2_DCFDA dye ruling out the autofluorescence (data not shown). These results suggest that SsnB compound promotes intracellular ROS production in neuroblastoma cell lines. We further evaluated SsnB-induced generation of ROS by fluorometry assay. Neuroblastoma cells as treated above were incubated with H_2_DCFDA dye for 30 min and the fluorescence intensity was measured at 485 nm (excitation wavelength) and 530 nm (emission wavelength). The H_2_DCFDA fluorescence intensity (as measure of generation of ROS) was significantly increased in cells treated with SsnB (10 µM) compared to DMSO control (*p<0.05, SsnB 10 µM *vs* DMSO; Figure 2C) whereas the H_2_DCFDA fluorescence intensity levels were almost similar in SsnB (1 µM) and DMSO-treated cells (ns  =  nonsignificant, SsnB 1 µM *vs* DMSO; Figure 2C).

In a reaction system, after the removal of acetate group by cellular esterases and oxidation by reactive oxygen species, the non-fluorescent carboxy-H_2_DCFDA dye is oxidized to fluorescent molecule carboxy-DCF. To measure the ROS-generating activity of SsnB, we also monitored the oxidation of carboxy-H_2_DCFDA dye in a cell-free system containing carboxy-H_2_DCFDA dye, horseradish peroxidase (HRP) and SsnB at 540 nm [38]. HRP was used to supply esterase since without esterase carboxy-H_2_DCFDA is not sensitive to oxidant. As shown in Figure 2D, the optical density (OD) value of reaction containing SsnB (10 µM) + H_2_DCFDA dye (25 µM) remained constant throughout the experiment suggesting that SsnB (10 µM) alone was not able to oxidize H_2_DCFDA to the fluorescent compound carboxy-DCF. After addition of HRP (5 units/ml) in the reaction mixture containing SsnB (10 µM) + H_2_DCFDA dye (25 µM), the OD value increased by 2% in the first 240 sec and further increased by 8% after 15 min compared to H_2_DCFDA + HRP reaction, indicating the increased oxidation of H_2_DCFDA to the fluorescent compound. These results suggest that SsnB in presence of cellular peroxidases oxidizes non-fluorescent H_2_DCFDA dye into the fluorescent compound. Addition of GSH (0.25 µM or 2.5 µM) in a reaction containing SsnB (10 µM) + H_2_DCFDA dye (25 µM) + HRP (5 units/ml) lowers the OD value in a dose dependent manner indicating that GSH as an antioxidant removes the free radicals generated by SsnB in reaction mixture which in turn inhibits the oxidation H_2_DCFDA dye (Figure 2E).

At cellular level, reduced glutathione (GSH) protects cells from oxidative damage resulted from increased superoxides, peroxides and free radicals [39]. Since SsnB induces oxidative stress, we asked whether SsnB affects intracellular levels of GSH in neuroblastoma cells. After treatments with SsnB for 2 days (SH-SY5Y and IMR-32), 3 days (NGP cells) or 4 days (SKNF-1 and SK-N-BE(2) cells), the cellular GSH level was measured by luminescence based glutathione assay kit. Compared to control, the GSH level was significantly decreased (∼40% of control) in cells treated with 10 µM SsnB (*p<0.05, SsnB 10 µM *vs* control; Figure 2F). However, the level of GSH at 1 µM SsnB concentration was slightly higher or equals to control levels (#*p*≤0.05 *vs* control) suggest that at initial stage basal antioxidant mechanism trying to protect the cell against the toxic effects of SsnB. These results indicated that the GSH depletion was unable to protect neuroblastoma cells against SsnB (10 µM)-induced increased oxidative stress.

### SsnB Promotes DNA Fragmentation in Neuroblastoma Cells

**Figure 3:**
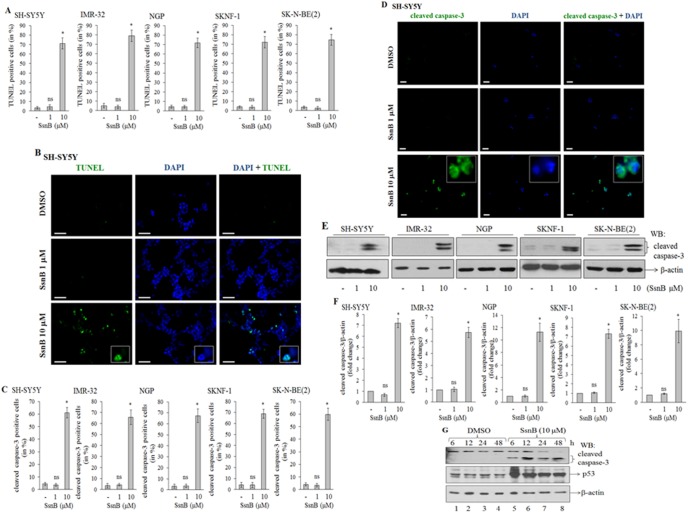
SsnB induces apoptotic cell death. SsnB-treated neuroblastoma cells (SH-SY5Y and IMR-32 for 2 days, NGP for 3 days, and SKNF-1 and SK-N-BE(2) cells for 4 days) were used to monitor DNA fragmentation by TUNEL assay (A and B), and cleaved caspsase-3 by immunofluorescence (C and D), and Western blotting (E and F). DAPI (blue) was used to stain nuclei. Signals were examined under fluorescence microscope; number of TUNEL-positive cells (green) or cleaved caspase-3 positive cells (green) were counted by ImageJ programme and plotted (**A and C**). Bar represents mean and S.D. of three independent experiments and *p<0.05 *vs* control. ns  =  nonsignificant *vs* control. (**B**) Representative images from SH-SY5Y TUNEL-assay showing that the number of TUNEL-positive cells (green, as indicator of DNA fragmentation) were more in SsnB-treated groups (10 µM) compared to DMSO or SsnB (1 µM). The enlarged overlapped and individual images showing cells with a typical apoptotic nucleus as stained with DAPI (blue) and TUNEL (green), and TUNEL staining is observed in those cells which had fragmented nuclei. Scale bar = 100 µm. (**D**) Representative immunofluorescence images showing staining for cleaved caspase-3 (green) in SH-SY5Y cells. Enlarged single and overlapped images showing that cleaved caspase-3 staining is exclusively present in the cytoplasm. Scale bar = 100 µm. (**E**) Western blot for cleaved caspase-3. Total cell extracts prepared from neuroblastoma cells treated with or without SsnB (1 µM or 10 µM) were separated on 14% SDS-polyacylamide gel and Western blotted with cleaved caspase-3 antibody. β-actin was used to check loading differences. Protein signal intensities were calculated form ImageJ programme and the ratio of cleaved caspase-3/β-actin were plotted (**F**). Bar represents mean and S.D. of three independent experiments and *p<0.05, SsnB 10 µM *vs* control; ns  =  nonsignificant *vs* control. (**G**) Western blots showing protein expression level for cleaved caspase-3 and p53 in total SH-SY5Y cell extract prepared from SsnB treatment (10 µM) at different time points (6, 12, 24, 48 h). β-actin was used to check loading differences.

Increased levels of reactive oxygen species (ROS) are known to induce oxidative stress which in turn cleaves DNA that leads to cell death. We next examined SsnB-induced DNA fragmentation in neuroblastoma cells by terminal deoxynucleotidyl transferase dUTP nick end labeling (TUNEL) assay as described previously [32]. In this assay, nicked DNA is identified by the addition of fluorophore-labelled dUTPs by enzyme terminal deoxynucleotidyl transferase (TdT) to the terminal ends of damaged DNA in apoptotic cells. The TUNEL-positive cells (green) were quantitated by ImageJ programme and plotted. The number of TUNEL-positive cells at 10 µM concentration of SsnB were significantly higher in all neuroblastoma cells compared to control (*p<0.005 *vs* control; Figure 3A). The TUNEL-positive cells in SsnB (1 µM) were almost similar to DMSO-treated samples (ns  =  nonsignificant *vs* control, *p*>0.05). The representative fluorescence images from SH-SY5Y cells as shown in Figure 3B indicated that SsnB at 10 µM concentration induced DNA fragmentation (green), and the staining for fragmented DNA (TUNEL-positive staining, green in color) and nuclei (blue) were overlapped in a single cell (as shown in enlarged image) suggest that SsnB promotes apoptosis in these cells. However, such TUNEL-positive cells were not detected in DMSO- or SsnB (1 µM)-treated cells.

### SsnB Activates Caspase-3 in Neuroblastoma Cells

At cellular level, mitochondria- or receptor (Fas/FasL)-mediated pathways activate caspase signaling cascade which in turn induces DNA damage and initiates programmed cell death (apoptosis) [40]. To determine whether SsnB-induced cell death is mediated by caspase activation, we performed Western blotting as well as immunofluorescence assay to detect active form of caspase-3 in neuroblastoma cells treated with SsnB for 2 days (SH-SY5Y and IMR-32 cells), 3 days (NGP cells) or 4 days (SKNF-1 and SK-N-BE(2) cells). The number of cleaved caspase-3 positive cells (green) were quantitated by ImageJ programme and plotted. The bar diagram in Figure 3C demonstrated that SsnB at 10 µM significantly increased the cleaved caspase-3 positive cells (green) compared to DMSO or SsnB (1 µM) treated neuroblastoma cells (*p<0.005, SsnB 10 µM *vs* control). The representative immunofluorescence images for SH-SY5Y in Figure 3D indicated that the color intensity of cleaved caspase-3 (green) was significantly increased in SsnB-treated (10 µM) cells and the cleaved caspase-3 fluorescence signals were exclusively present in cytoplasm and did not overlap with nucleus (blue). The corresponding Western blots in Figure 3E demonstrated that the cleaved and active form of caspase-3 protein bands (17 and 19 kDa) were present in protein samples prepared from 10 µM SsnB treated cells (*p<0.005, SsnB 10 µM *vs* control, Figure 3E and F), while proteins samples from DMSO and SsnB (1 µM) had almost undetectable levels of cleaved caspase-3 (ns  =  nonsignificant, SsnB 1 µM *vs* control; Figure 3E and F). We further examined the activation of caspase-3 by SsnB (10 µM) at different time points (6, 12, 24 and 48 h) in SH-SY5Y cells. The presence of an active form of caspase-3 protein band (∼19 kDa) showed that SsnB induces cleavage of caspase-3 in these cells even as early as 6 h SsnB exposure (Figure 3G, upper panel). Thus, these data suggest that SsnB induces apoptosis through activation of caspase-3 in all neuroblastoma cell lines tested. We also observed increased expression of p53 in SsnB (10 µM) treated cells compare to DMSO-treated cells (middle panel; Figure 3G). Activation of p53, a tumor suppressor protein, leads to growth arrest at G1 or G2 phase of cell cycle, hence, increased expression of p53 by SsnB suggested that p53 might also involve in SsnB-induced cytotoxicity in p53-containing neuroblastoma cells.

### SsnB Reduces Expression of N-myc in Neuroblastoma Cells

**Figure 4:**
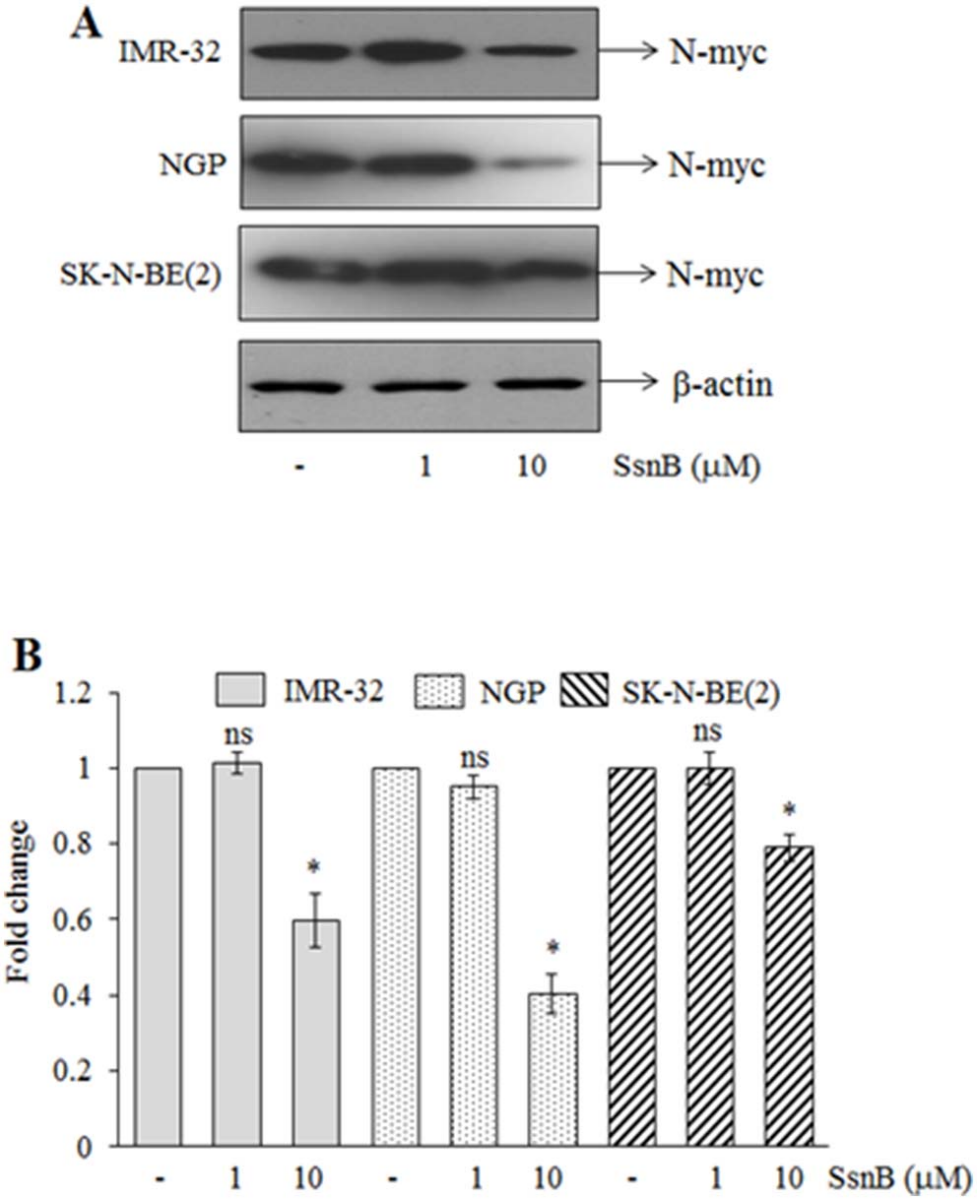
N-myc protein level in IMR-32, NGP and SK-N-BE(2) cells. (A) Representative Western blots for N-myc protein in total cell extract prepared from IMR-32, NGP and SK-N-BE(2) cells treated with or without SsnB (1 µM or 10 µM) for 2 days, 3 days or 4 days, respectively. β-actin was used to check loading differences. (**B**) Bar diagram represents the fold change in N-myc protein signals as measured by imageJ programme. Bar represents mean and S.D. of three independent experiments and *p<0.05, SsnB 10 µM *vs* control; ns  =  nonsignificant *vs* control.

N-myc proto-oncogene act as a transcription factor, and amplification and overexpression of N-myc gene promotes tumorigenicity in neuroblastoma cells [41]. We evaluated level of N-myc proteins from 2 days (IMR-32), 3 days (NGP cells) or 4 days (SK-N-BE(2) cells) SsnB-treated N-myc amplified neuroblastoma cells by Western blotting, and protein signal intensity was measured by ImageJ programme and plotted. Western blot in Figure 4A demonstrates that SsnB at 10 µM inhibited the expression of N-myc protein in these cell lines (*p<0.005, SsnB 10 µM *vs* control; Figure 4A and B). However such reduction in protein expression was not observed at 1 µM concentration of SsnB (ns  =  nonsignificant, SsnB 1 µM *vs* control; Figure 4A and B).

### N-acetylcystamine (NAC) Attenuates the Inhibitory Effects of SsnB in Neuroblastoma Cells

**Figure 5:**
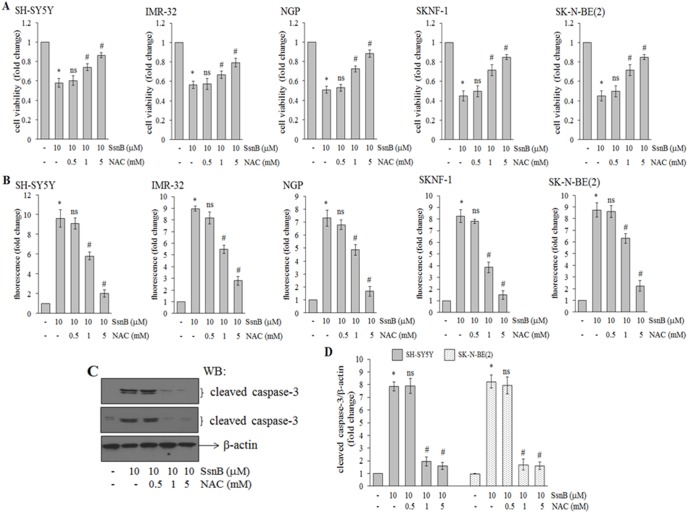
N-acetylcysteine (NAC) attenuates the inhibitory effects of SsnB. (A) Neuroblastoma cells were pretreated with NAC (0.5, 1 or 5 mM) for 1 h followed by SsnB (10 µM) treatment for 2 days (SH-SY5Y and IMR-32), 3 days (NGP) or 4 days (SKNF-1 and SK-N-BE(2) cells) and after treatments MTT cell viability assays was carried out. Bar represents mean and S.D. of three independent experiments and *p<0.05 *vs* control; ns  =  nonsignificant *vs* SsnB 10 µM; #*p*<0.05 *vs* SsnB 10 µM. (**B**) Bar diagram representing the fold change in H_2_DCFDA-fluorescence intensity in cells treated with SsnB (10 µM) alone or in combinations with NAC (0.5, 1 or 5 mM) as mentioned above. After labelling with H_2_DCFDA dye (25 µM) for 30 min fluorescence intensity was monitored at excitation wavelength 485 nm and emission wavelength 530 nm. Bar represents mean and S.D. of three independent experiments and *p<0.05 *vs* control; ns  =  nonsignificant *vs* SsnB 10 µM; #*p*<0.05 *vs* SsnB 10 µM. (**C**) Representative Western blots demonstrating level of cleaved caspase-3 in total cell protein isolated from SH-SY5Y (upper panel) and SK-N-BE(2) cells (lower panel) treated with NAC (0.5, 1 or 5 mM) and SsnB (10 µM) alone or in combinations for 2 days and 4 days, respectively. β-actin was used to check loading differences. Bar diagram represents the fold change in protein levels as measured by imageJ programme (**D**). Bar represents mean and S.D. of three independent experiments and *p<0.05, SsnB 10 µM *vs* control; ns  =  nonsignificant *vs* control; #*p*<0.05 *vs* SsnB 10 µM.

The reduced cell viability, increased ROS and decreased GSH levels by SsnB indicated that depletion of glutathione may be a primary cause for the cell death. Next, we examined whether addition of N-acetylcystamine (NAC), a GSH precursor and an antioxidant, can protect neuroblastoma cells from SsnB-induced cell death [42]. To test this hypothesis, neuroblastoma cells were pretreated with NAC (0.5, 1 and 5 mM) for 1 h followed by treatment with SsnB (10 µM) for 2 days (SH-SY5Y and IMR-32 cells), 3 days (NGP cells) or 4 days (SKNF-1 and SK-N-BE(2) cells), and cell viability was measured by MTT assay. MTT cell viability assays showed that NAC treatment suppresses SsnB-induced cell death in a concentration dependent manner (Figure 5A). SsnB (10 µM) induced neuroblastoma cell death in range of 40–60% (*p<0.05 *vs* control) whereas presence of NAC (1 mM or 5 mM) significantly increased the viability of SsnB-treated cells (#*p*<0.05, SsnB 10 µM alone *vs* SsnB 10 µM + NAC 1 or 5 mM). Next we measured the levels of ROS in SsnB-treated cells preincubated with NAC by fluorometry assay using H_2_DCFDA dye. NAC at 1 and 5 mM concentration significantly reduces ROS levels in SsnB (10 µM) treated neuroblastoma cells (#*p*<0.05, SsnB 10 µM alone *vs* SsnB 10 µM + NAC 1 or 5 mM; Figure 5B). These data suggest that NAC as an ROS scavenger removes SsnB-induced generation of reactive oxygen species in cells.

We further examined cleaved caspase-3 level in SH-SY5Y cells (2 days treatment) and SK-N-BE(2) cells (4 days treatment) incubated with SsnB (10 µM) with or without NAC (0.5, 1 or 5 mM) by Western blotting. Representative blot in Figure 5C and corresponding bar diagram (Figure 5D) demonstrated that pretreatment of NAC (1 and 5 mM) to SsnB treated cells inhibited the activation on caspase-3 and brings to control levels in both SH-SY5Y (upper panel) and SK-N-BE(2) cells (middle panel) (#*p*<0.05, SsnB 10 µM *vs* SsnB 10 µM + NAC 1 or 5 mM).

### SsnB Reduces the Tumorigenicity of Neuroblastoma Cells

**Figure 6:**
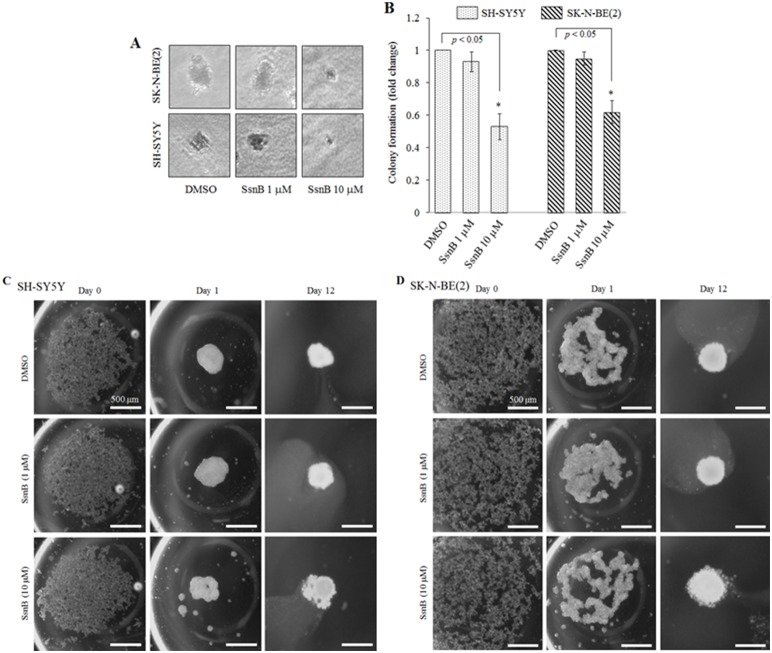
SsnB reduces the tumorigenicity of neuroblastoma cells. Phase contrast images showing neuroblastoma colonies formed after treatment with SsnB (1 and 10 µM) for 45 days in anchorage-independent agarose gel colony formation assay (**A**). The number of colonies was counted and plotted (**B**). Bar represents mean and S.D. of three independent experiments and *p<0.05 was considered significant. (**C and D**) SsnB inhibits the neuroblastoma spheroid formation. Phase contrast images showing spheroid formation in absence or presence of SsnB (1 or 10 µM) in 3-D hanging drop assay. Twenty microliter drop containing 20,000 cells (SH-SY5Y, **C**; and SK-N-BE(2) cells, **D**) were pipetted on the lower side of the lid of petri dish. The lid was gently inverted, placed on top of the petri dish and let them grow at 37°C. On day 0, day 1 and day 12 the image of cells in each droplet was taken by Olympus inverted microscope to monitor the spheroid formation. Scale bar = 500 µm.

Next we tested if the anti-proliferative activity of SsnB affects the tumor progression property of neuroblastoma cells by performing two layer agarose gel colony formation assays. Agarose gel colony formation assay is an anchorage-independent *in vitro* cell transformation assay, and mimics the process of *in vivo* carcinogenesis [34]. Neuroblastoma cells (N-myc amplified cell, SK-N-BE(2), and N-myc nonamplified cell, SH-SY5Y) were grown in agarose in presence or absence of SsnB (1 µM and 10 µM) and after 45 days number of colonies were counted. The representative phase contrast images in Figure 6A showed that the size of colonies in SsnB 10 µM treated samples were significantly smaller than the control and SsnB (1 µM) treated samples. The representative bar diagram demonstrated that SsnB at 10 µM concentration significantly inhibited the colony formation capability of both cells (*p<0.05, SsnB 10 µM *vs* control; Figure 6B).

Since SsnB inhibits the formation of neuroblastoma colony in anchorage-independent agarose gel assay, we further tested if the inhibitory effects of SsnB inhibit the initiation of neuroblastoma tumors in hanging drop assay method. In hanging drop assay the cells under the force of gravity and surface tension start accumulating at the bottom of the hanging drop and join together to form a spheroid. Spheroids represent an *in vitro* 3D tissue structure that mimics *in vivo* tumor tissue organization and microenvironment, and 3D culture better reflect cancer cells in their native, *in vivo,* environment [35]. As shown in Figure 6C and D, within 24 hour, both neuroblastoma cells (SH-SY5Y cells, C; and SK-N-BE(2) cells, D) grown in hanging drop culture start aggregating at the bottom of the droplet. On day 12, the shape of the aggregates became rounder and smoother with a gradual decrease in the radius of the spheroid, and form compact cell clusters in DMSO- and SsnB (1 µM)-treated SH-SY5Y and SK-N-BE(2) cells. The decrease in size is the result of higher cell–cell cohesion, which yields more compact aggregates. However, at 10 µM SsnB concentration the cells are loosely attached in spheroids suggesting that SsnB at 10 µM inhibits cell-cell attachment in both cell lines (SH-SY5Y and SK-N-BE(2) cells) as a result these cells are unable to aggregate to form compact spheroids.

## Discussion

Neuroblastoma has heterogeneous population of cells of different genetic background [15]. Increasing evidence supports that molecular and genetic factors such as N-myc oncogene amplification, deletion of short arm of chromosome 1 and high expression of neurotrophin receptors (TrkA and TrkB) are associated with malignant transformation and progression of neuroblastoma. Despite targeting new molecular targets, and the use of multimodal therapy which includes surgery, radiotherapy in conjunction with chemotherapy and monoclonal antibody based immunotherapy, approximately 40% of children with high-risk neuroblastoma remain incurable [23], [43], [44]. Hence, the identification and development of new therapeutic compounds with less toxicity are urgently needed.

We used a novel naturally-occurring compound, Sparstolonin B (SsnB), isolated from the tubers of an aquatic Chinese herb, *Sparganium stoloniferum* [1]. The crude extract prepared from this herb has anti-spasmodic and anti-tumor properties [4]–[6]. In the present study SsnB at 10 µM and above concentrations significantly inhibits the growth and viability of human neuroblastoma cells of different genetic background such as N-myc amplified with wild p53 cells (IMR-32 and NGP cells), N-myc amplified with mutated p53 cells (SKN-BE(2) cell), and N-myc nonamplified cell (SH-SY5Y and SKNF-1 cells). However, the sensitivity of these cells towards SsnB is different, SH-SY5Y and IMR-32 cells are sensitive, NGP cells are moderate sensitive, and SKNF-1 and SK-N-BE(2) cells are least sensitive (as shown by cell morphology and MTT cell viability assays in Figure 1A and B). The reduced viability by SsnB losses neuroblastoma cells ability to form compact spheroids that decreases the tumorigenic potential of both N-myc amplified (SK-N-BE(2) cells) and N-myc nonamplified neuroblastoma cells (SH-SY5Y cells) tested as seen in hanging drop method and anchorage-independent colony formation assay (Figure 6). These *in vitro* 3-D culture results further confirmed the anti-tumorigenic activity of SsnB against human neuroblastoma. The SsnB exerts its inhibitory effects only in neuroblastoma cell lines, and up to ∼100 µM concentration SsnB did not exhibits cytotoxicity towards various normal non-tumor human cells such as human monocytic THP-1 cells, phorbol 12-myristate 13-acetate-differentiated THP-1 macrophages, human umbilical vein endothelial cells and human aortic smooth muscle cells [1].

The inhibitory effects of SsnB on cell growth and viability are resulted from the caspase-mediated cell death (Figure 3C–F). The increased level of activated form of caspase-3 has been shown to trigger DNA fragmentation, chromatin condensation, membrane blebbing and cell shrinkage that leads to the programme cell death called apoptosis [40]. The presence of apoptotic nuclei in SsnB-treated neuroblastoma cells compared to control DMSO-treated cells (as revealed by TUNEL assay, Figure 3A and B) indicates that activation of caspase cascade by SsnB trigger apoptotic cell death pathways. In addition, we also found that SsnB promotes ROS generation in both N-myc amplified and N-myc nonamplified neuroblastoma cells (Figure 2A–C). The ROS generating capacity of SsnB was confirmed by our cell-free system in which SsnB in presence of peroxidase (HRP) oxidized H_2_DCDA to fluorescent compound (Figure 2D and E). Reactive oxygen species such as free radicals and peroxides increases cellular oxidative stress which promotes lipids peroxidation and oxidation of nucleic acid and amplifies the cell death. The imbalance between the generation of reactive oxygen species (ROS) and antioxidant defense status disrupt the redox homeostasis [39]. In our present study, we observed that SsnB significantly decreased glutathione (GSH) level in neuroblastoma cells (Figure 2F). GSH acts as an antioxidant and its role to remove oxidative stress caused by reactive oxygen species is well known hence GSH depletion in presence of SsnB fail to remove free radicals and this might be the primary cause of SsnB-induced cytotoxicity towards neuroblastoma cells. The critical role of GSH in SsnB-induced cytotoxicity was further supported by our observations obtained with N-acetylcystamine (NAC). NAC is a GSH precursor and functions as an antioxidant. Co-incubation of NAC removes free radicals from the SsnB-treated cells and inhibits the activation of cleaved caspase-3 (Figure 5B–D). These events thus protect the cells against the oxidative insults caused by SsnB and increased the cell viability (Figure 5A). Hence these data suggest that SsnB-induced cytotoxic effects are resulted by the excess generation of reactive oxygen species in neuroblastoma cells.

The induction of cell cycle arrest at a specific checkpoint and thereby inducing apoptosis is a common mechanism for the cytotoxic effects of anticancer drugs [45]. The cell cycle arrest at G2/M transition is a potential target for cancer therapy as it prevents DNA-damaged cells from entering mitosis, and cell cycle blockage at this checkpoint is carried out by cell cycle-related proteins such as p53 and cyclins. Activation of p53, a tumor suppressor protein, leads to growth arrest at G1 or G2 phase of cell cycle [46]. The results in present study demonstrate that treating neuroblastoma cells with SsnB (10 µM) resulted in cell cycle block in G2/M phase (flow cytometry analysis, Figure 1C) and increase in expression of p53 compared to DMSO treated cells (Figure 3G). These findings suggested that p53-mediated apoptosis might also involve in SsnB-induced cytotoxicity in p53-containing neuroblastoma cells.

It is well known that N-myc amplification is associated with neuroblastoma tumor progression and drug resistance [41], [47]. N-myc, a member of the myc family of proto-oncoprotein, acts as a transcription factor and regulates the expression of genes involved in cell cycle, DNA damage and apoptosis, and overexpression of N-myc in trasnsgenic mice results in neuroblastoma development [48]. However, downregulation of N-myc expression has been shown to induce growth arrest and apoptosis in neuroblastoma cells [49], [50]. In the current study, we found that SsnB at 10 µM concentration suppresses the N-myc expression in IMR-32, NGP and SK-N-BE(2) cells (Figure 4) which might be correlated with the SsnB-mediated anticancer activity on human neuroblastoma cells.

In summary, the present study for the first time demonstrated the anticancer property of SsnB on human neuroblastoma cells. SsnB induces apoptosis in a wide range of neuroblastoma cell lines. The reduced tumorigenic ability of both N-myc amplified and N-myc nonamplified cells resulted from SsnB in our colony formation and hanging drop assays, and a recent published study showing that SsnB exerts anti-angiogenic property by downregulating mRNA level of cell cycle regulatory proteins [3] indicated that SsnB can be a used as a novel therapeutics anticancer agent. Drugs that generate ROS and induce apoptosis are used alone or in combination with other therapies to treat cancer, suggesting that SsnB can be a promising antitumor agent for neuroblastoma and requires preclinical studies on animal models [51].
