# Correction: Sparstolonin B, a Novel Plant Derived Compound, Arrests Cell Cycle and Induces Apoptosis in N-Myc Amplified and N-Myc Nonamplified Neuroblastoma Cells

**DOI:** 10.1371/journal.pone.0159082

**Published:** 2016-07-06

**Authors:** Ambrish Kumar, Daping Fan, Donald J. DiPette, Ugra S. Singh

The authors would like to correct Fig 1, as errors were introduced in the preparation of this figure for publication. During the assembly of Fig 1A, the same image was inadvertently used for both the DMSO-treated SKNBE(2) cells (Column 1, Row 5) and SsnB 1 μM-treated SKNBE(2) (Column 2, Row 5) cells. The authors have provided a corrected version of Fig 1 here. The authors confirm that these changes do not alter their findings. The authors have provided the underlying images as Supporting Information.

**Fig 1:**
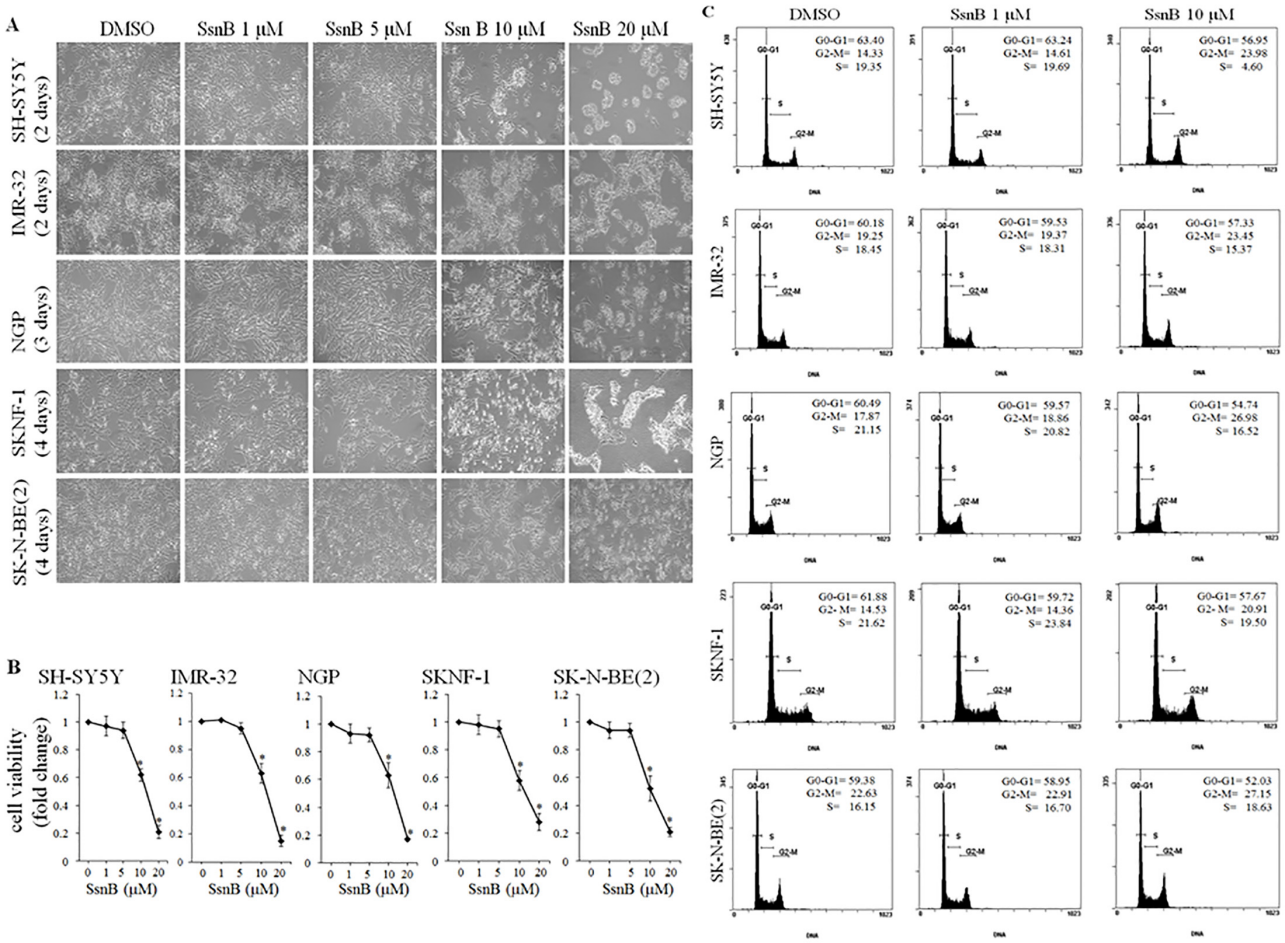
SsnB inhibits cell growth and viability of neuroblastoma cells. (A) Phase contrast images showing the morphology of neuroblastoma cells after treatments with SsnB. Neuroblastoma cells (SH-SY5Y, IMR-32, NGP, SKNF-1 and SK-N-BE(2) cells) were grown in presence of DMSO or SsnB (1, 5, 10, and 20 μM) in complete culture medium and were photographed on indicated times. (B) Bar diagrams showing the cell viability after SsnB treatment as evaluated by MTT assays. Neuroblastoma cells were treated with SsnB (1, 5, 10, and 20 μM) for 2 days (SH-SY5Y and IMR-32), 3 days (NGP) or 4 days (SKNF-1 and SK-N-BE(2) cells) and cell viability was measured by MTT assay at 575 nm. Data are represented in fold change and *p<0.05 vs control. (C) SsnB arrest cell cycle at G2/M phase. Representative histograms illustrating the cell cycle progression of neuroblastoma cells in presence of SsnB. Neuroblastoma cells treated with SsnB (1 μM or 10 μM) in DMEM with 10% FBS for 2 days (SH-SY5Y and IMR-32), 3 days (NGP) or 4 days (SKNF-1 and SK-N-BE(2) cells) were labelled with propidium iodide and cell cycle stage was analyzed by flow cytometry.

## Supporting Information

S1 File Raw images used to create Fig 1. (ZIP)

## References

[1] Kumar A, Fan D, DiPette DJ, Singh US (2014) Sparstolonin B, a Novel Plant Derived Compound, Arrests Cell Cycle and Induces Apoptosis in N-Myc Amplified and N-Myc Nonamplified Neuroblastoma Cells. PLoS ONE 9(5): e96343 doi: 10.1371/journal.pone.0096343 24788776 10.1371/journal.pone.0096343 PMC4006872

