# Supplementary figures and images for: Correction: Sparstolonin B, a Novel Plant Derived Compound, Arrests Cell Cycle and Induces Apoptosis in N-Myc Amplified and N-Myc Nonamplified Neuroblastoma Cells
Source: PLoS One. 2016 Jul 6;11(7):e0159082. doi: 10.1371/journal.pone.0159082 (PMC4934772; doi:10.1371/journal.pone.0159082)

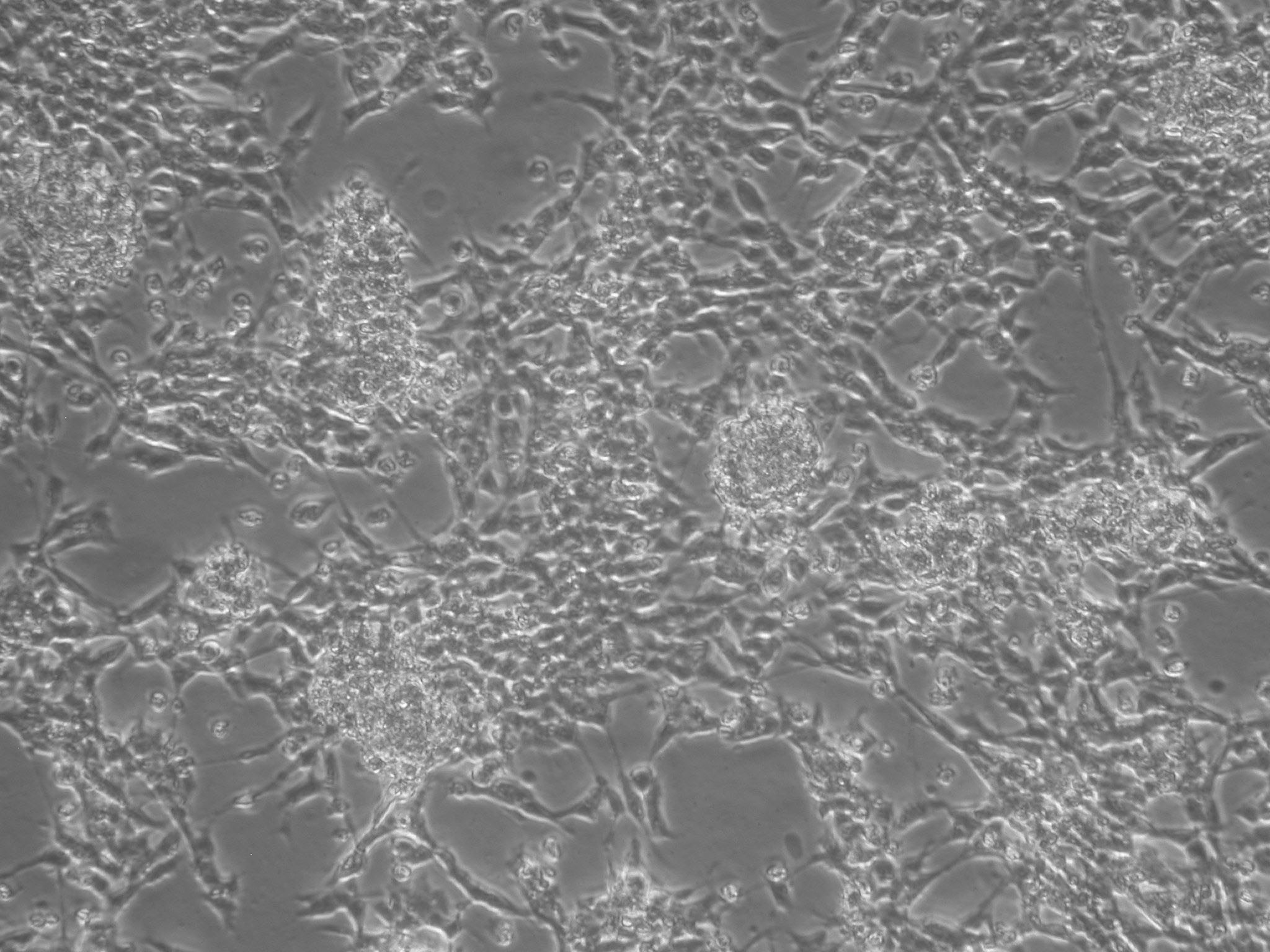

Supplement: S1 File — (ZIP) [file pone.0159082.s001.zip › S1 File/Fig 1A_IMR-32 DMSO.jpg]

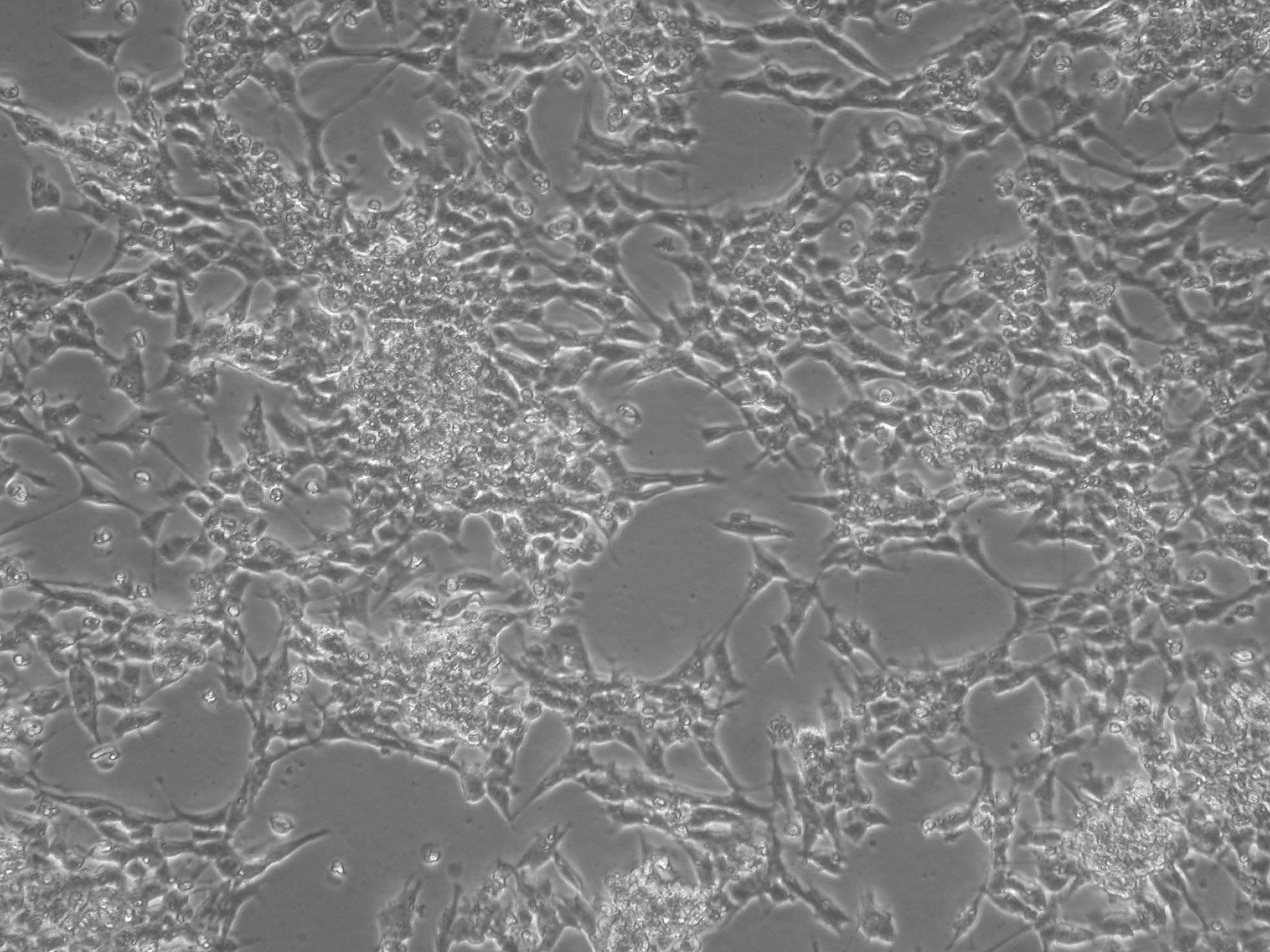

Supplement: S1 File — (ZIP) [file pone.0159082.s001.zip › S1 File/Fig 1A_IMR-32 SsnB 1 μM.jpg]

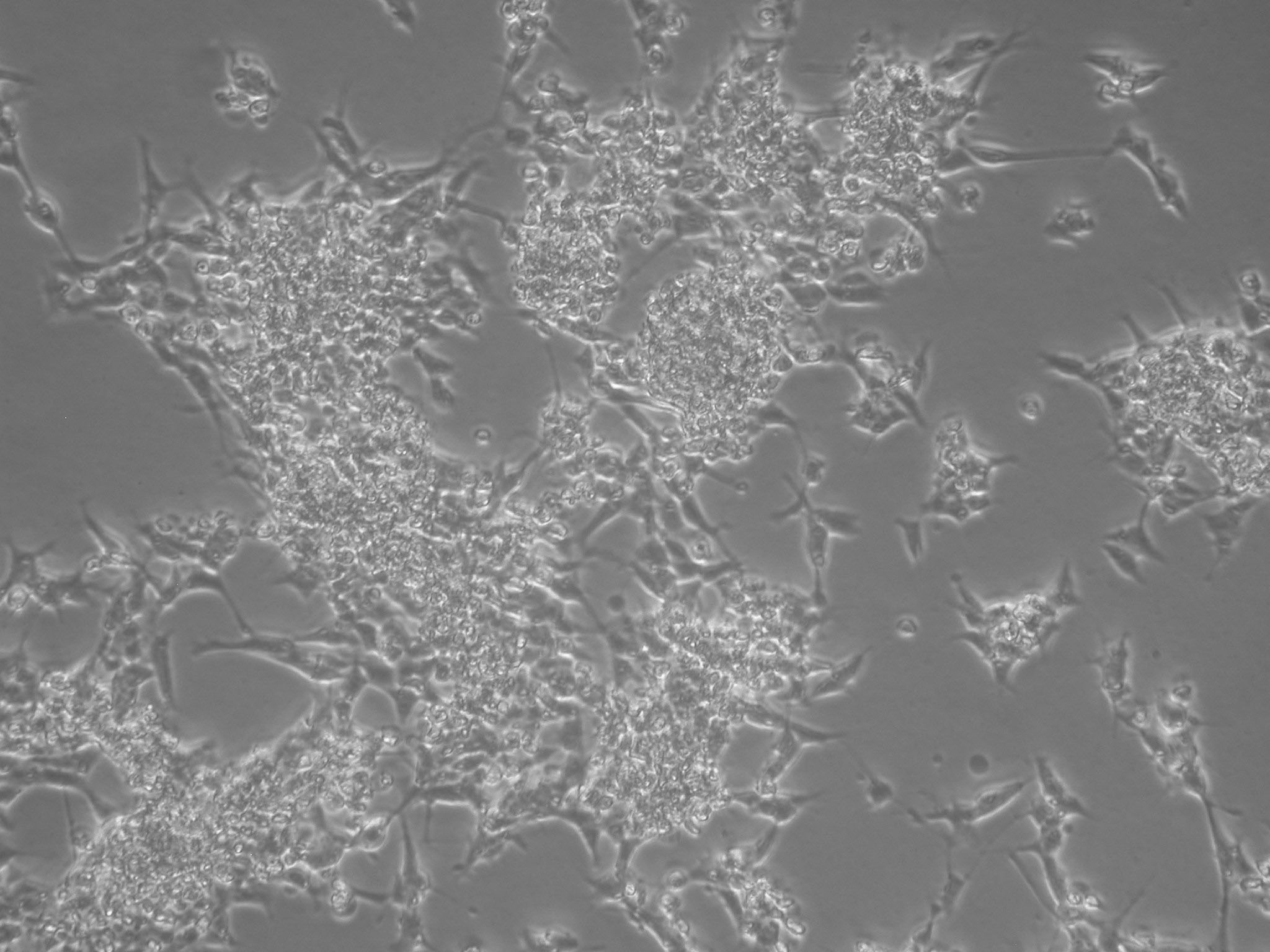

Supplement: S1 File — (ZIP) [file pone.0159082.s001.zip › S1 File/Fig 1A_IMR-32 SsnB 10 μM.jpg]

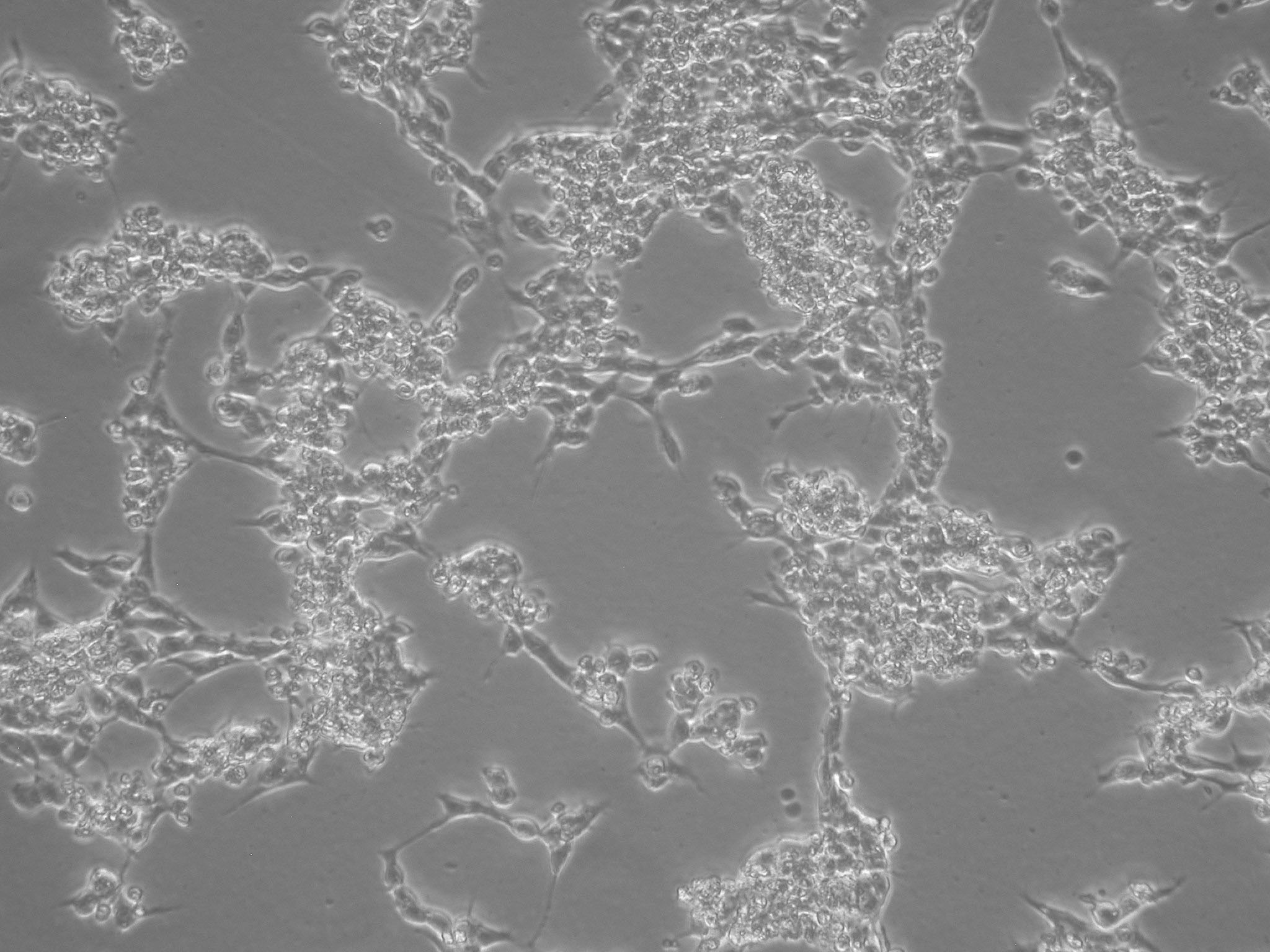

Supplement: S1 File — (ZIP) [file pone.0159082.s001.zip › S1 File/Fig 1A_IMR-32 SsnB 20 μM.jpg]

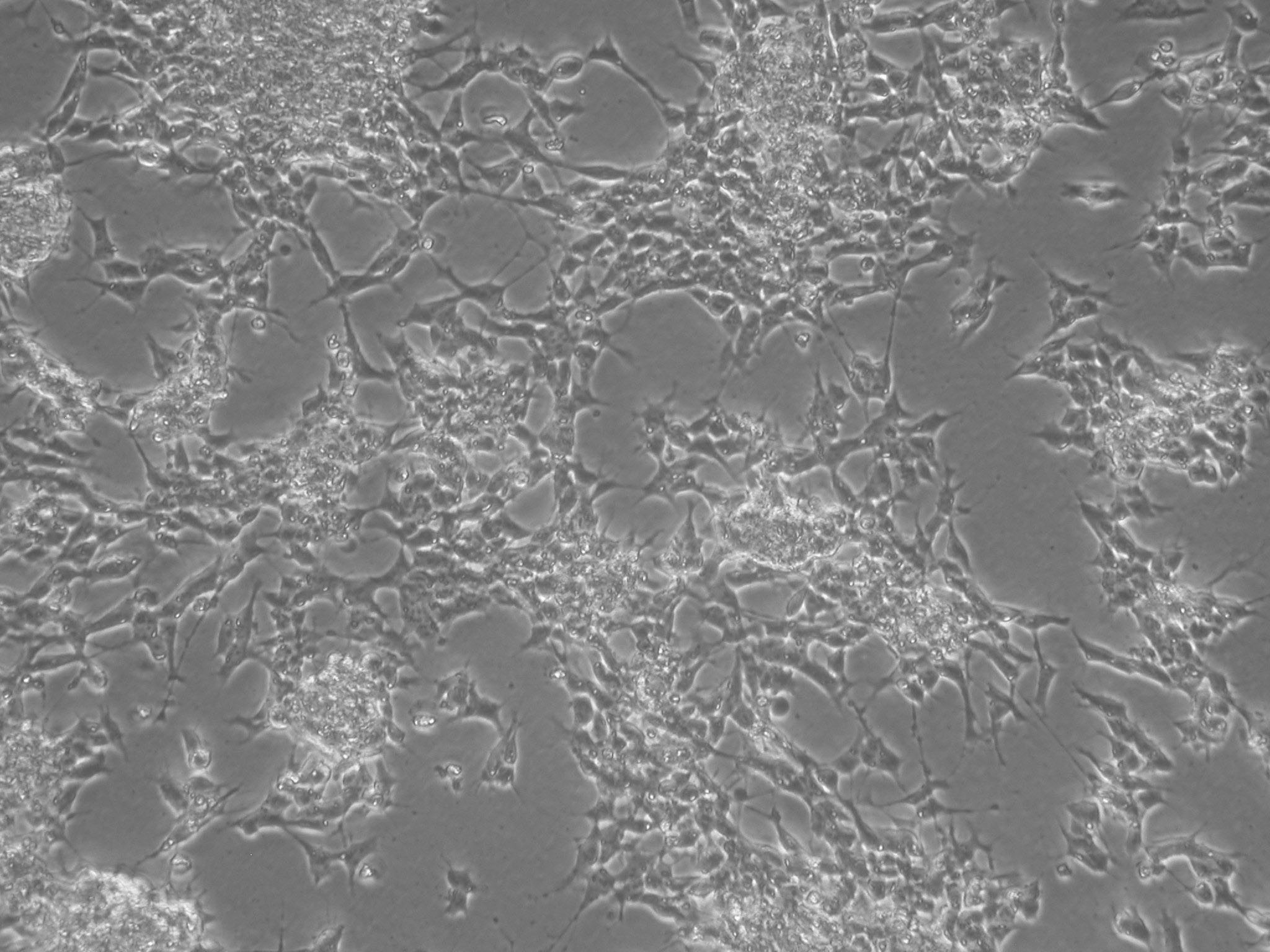

Supplement: S1 File — (ZIP) [file pone.0159082.s001.zip › S1 File/Fig 1A_IMR-32 SsnB 5 μM.jpg]

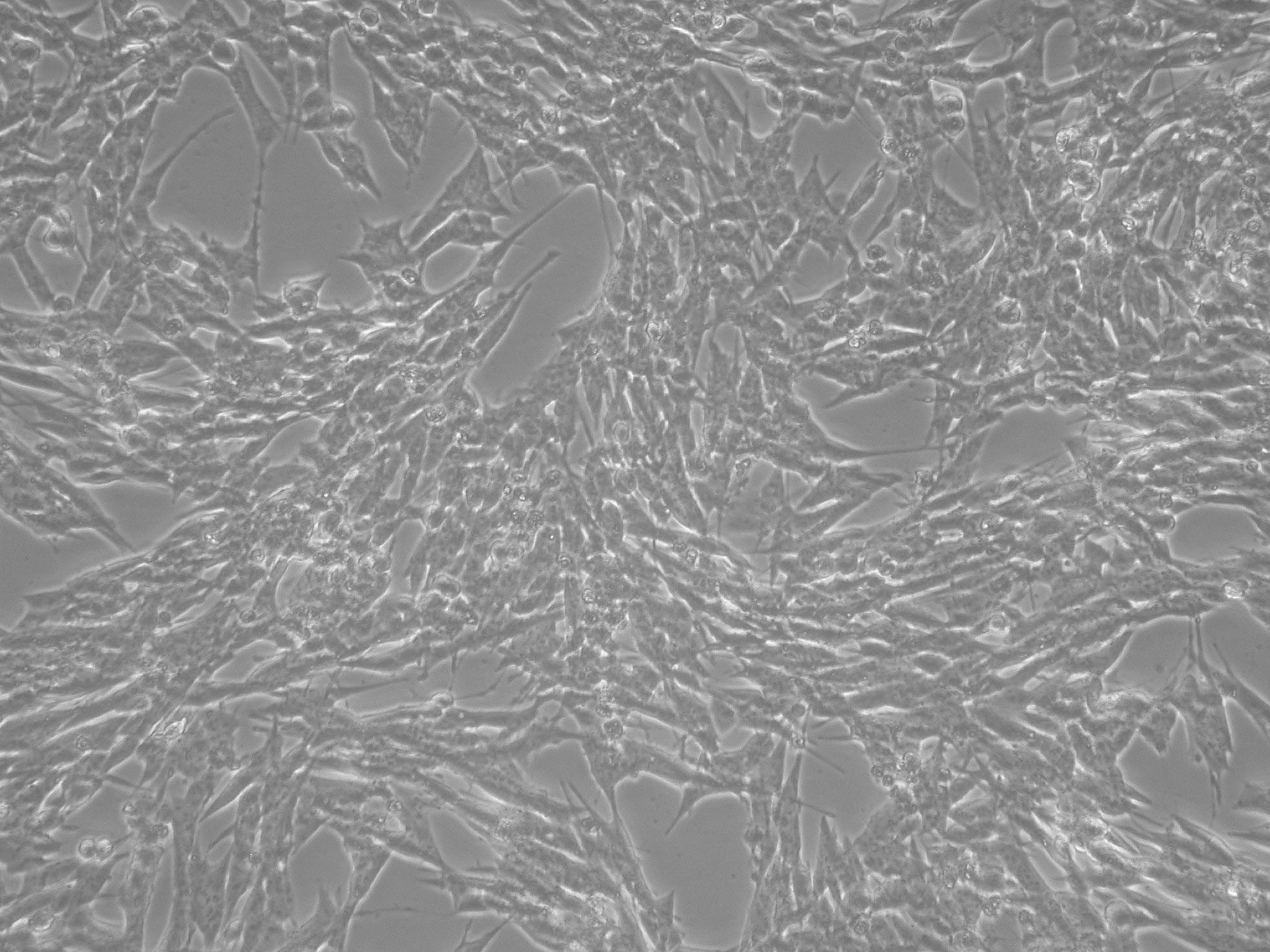

Supplement: S1 File — (ZIP) [file pone.0159082.s001.zip › S1 File/Fig 1A_NGP DMSO.jpg]

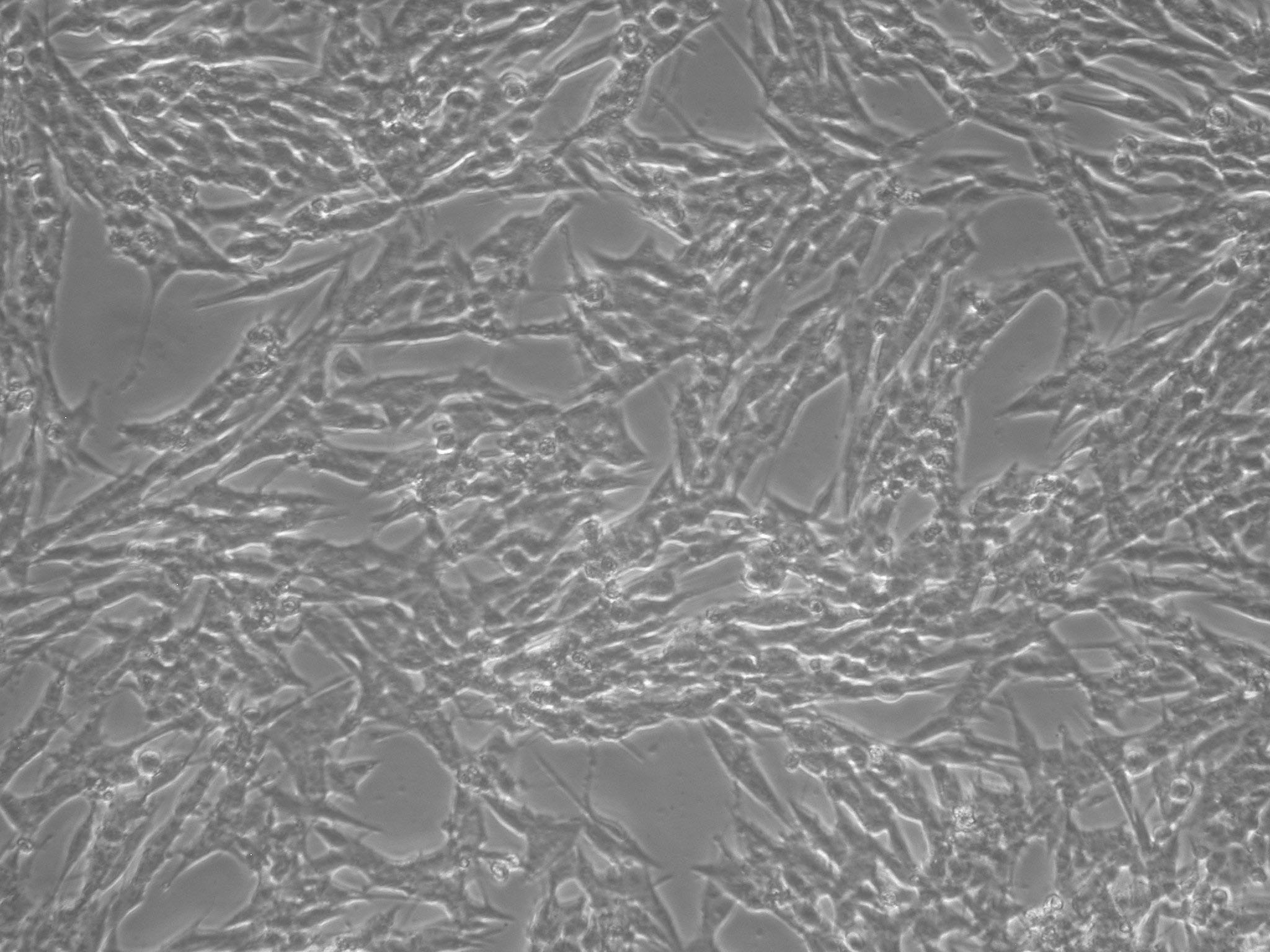

Supplement: S1 File — (ZIP) [file pone.0159082.s001.zip › S1 File/Fig 1A_NGP SsnB 1 μM.jpg]

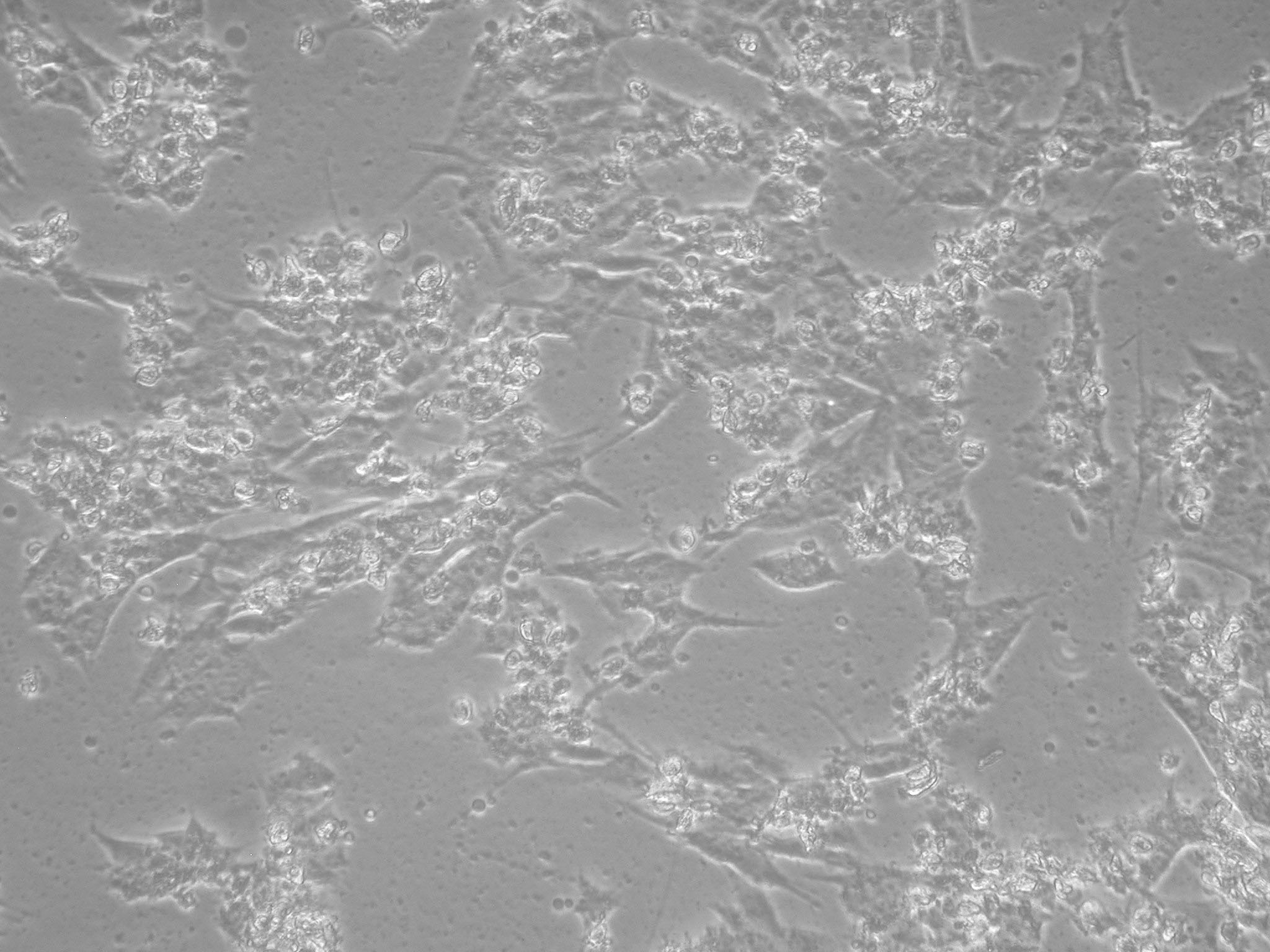

Supplement: S1 File — (ZIP) [file pone.0159082.s001.zip › S1 File/Fig 1A_NGP SsnB 10 μM.jpg]

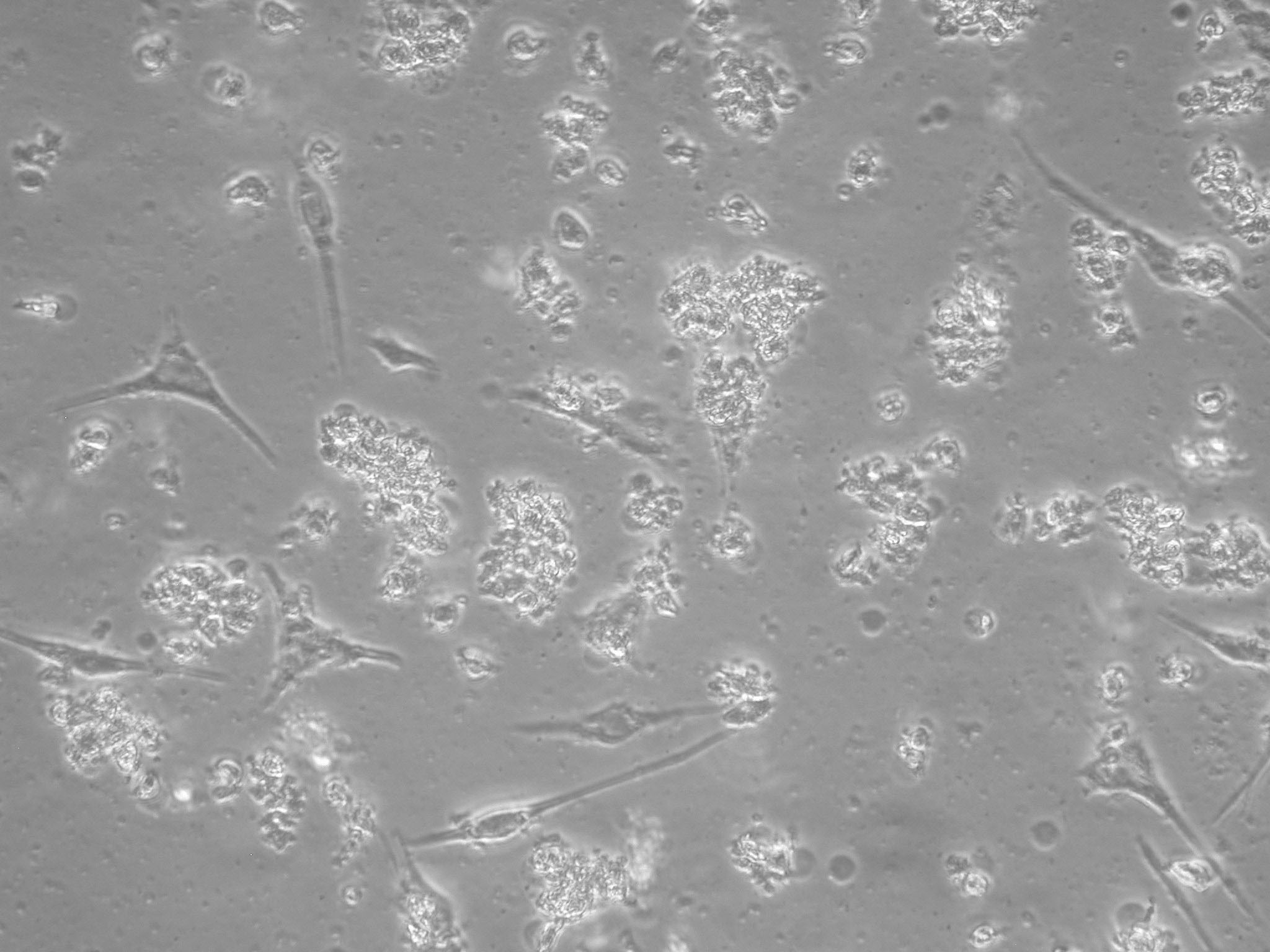

Supplement: S1 File — (ZIP) [file pone.0159082.s001.zip › S1 File/Fig 1A_NGP SsnB 20 μM.jpg]

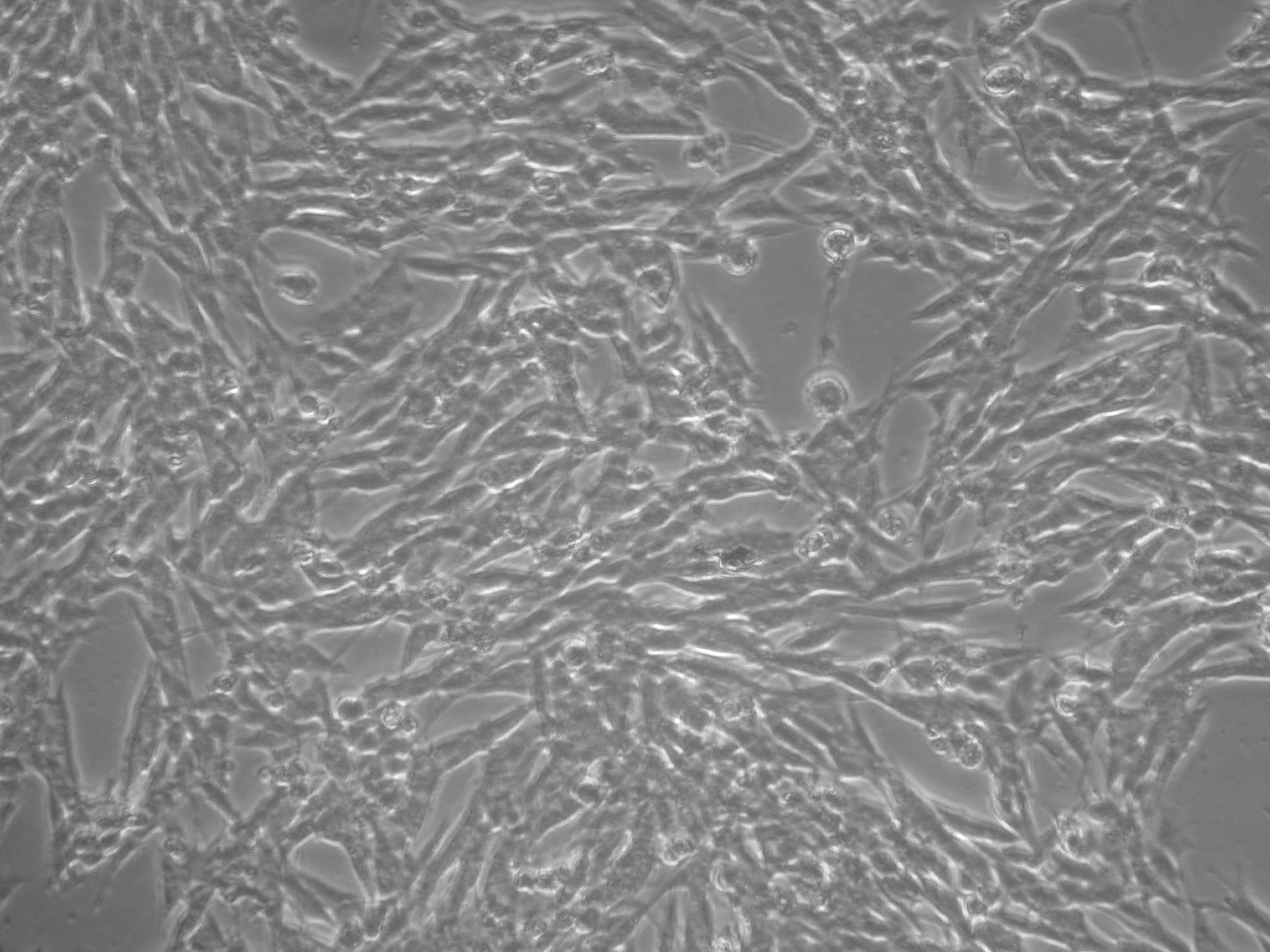

Supplement: S1 File — (ZIP) [file pone.0159082.s001.zip › S1 File/Fig 1A_NGP SsnB 5 μM.jpg]

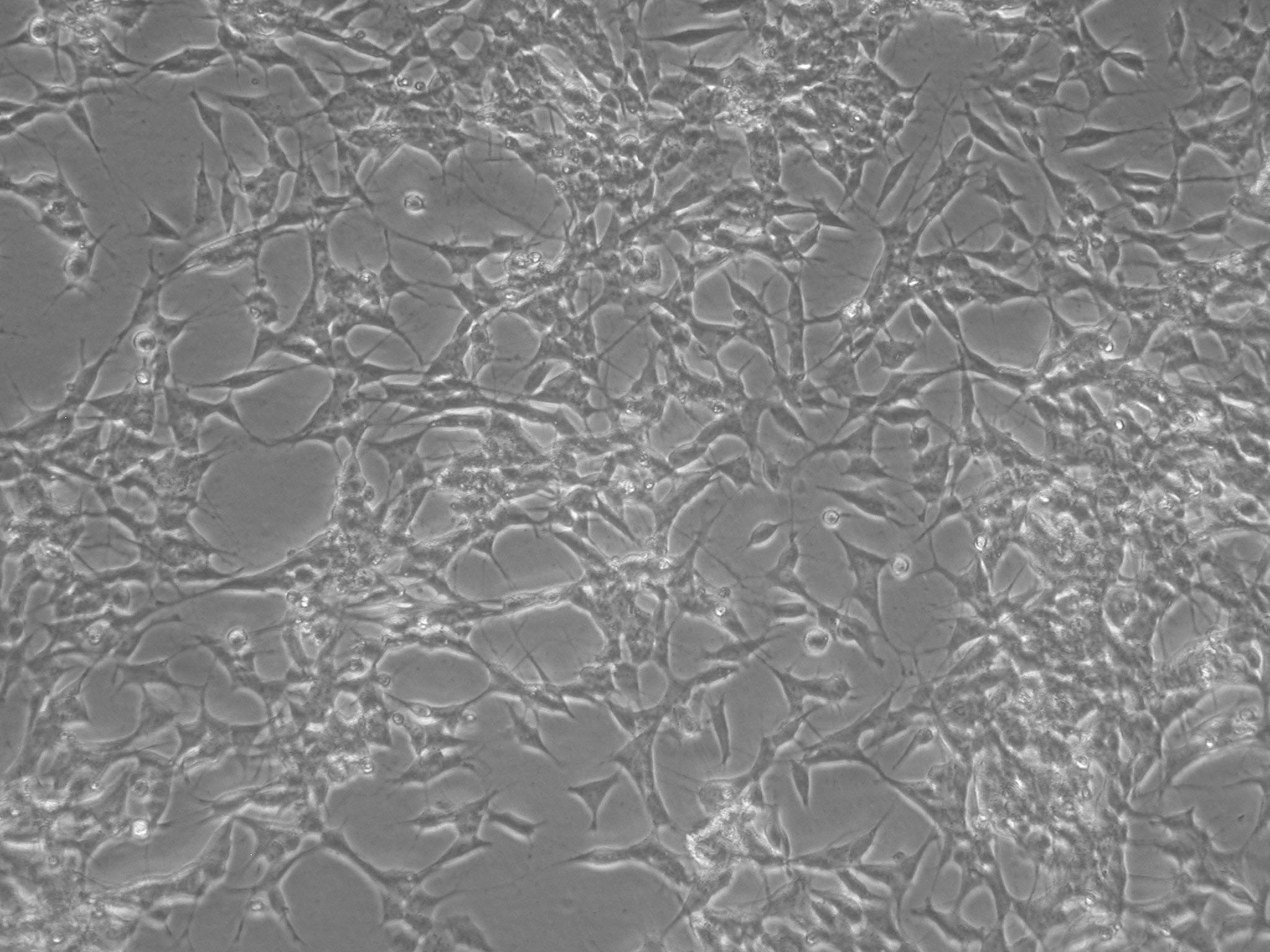

Supplement: S1 File — (ZIP) [file pone.0159082.s001.zip › S1 File/Fig 1A_SH-SY5Y DMSO.jpg]

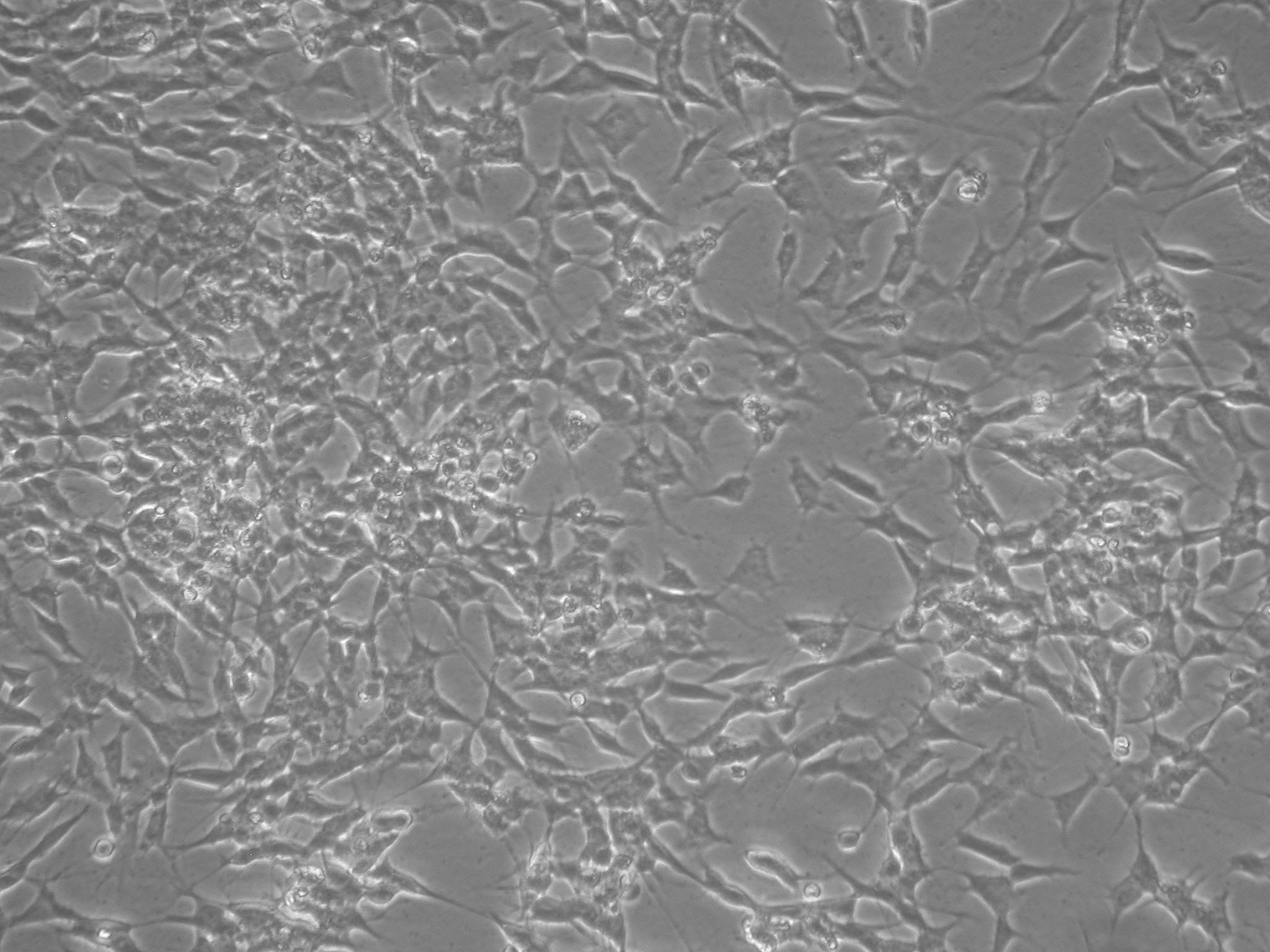

Supplement: S1 File — (ZIP) [file pone.0159082.s001.zip › S1 File/Fig 1A_SH-SY5Y SsnB 1 μM.jpg]

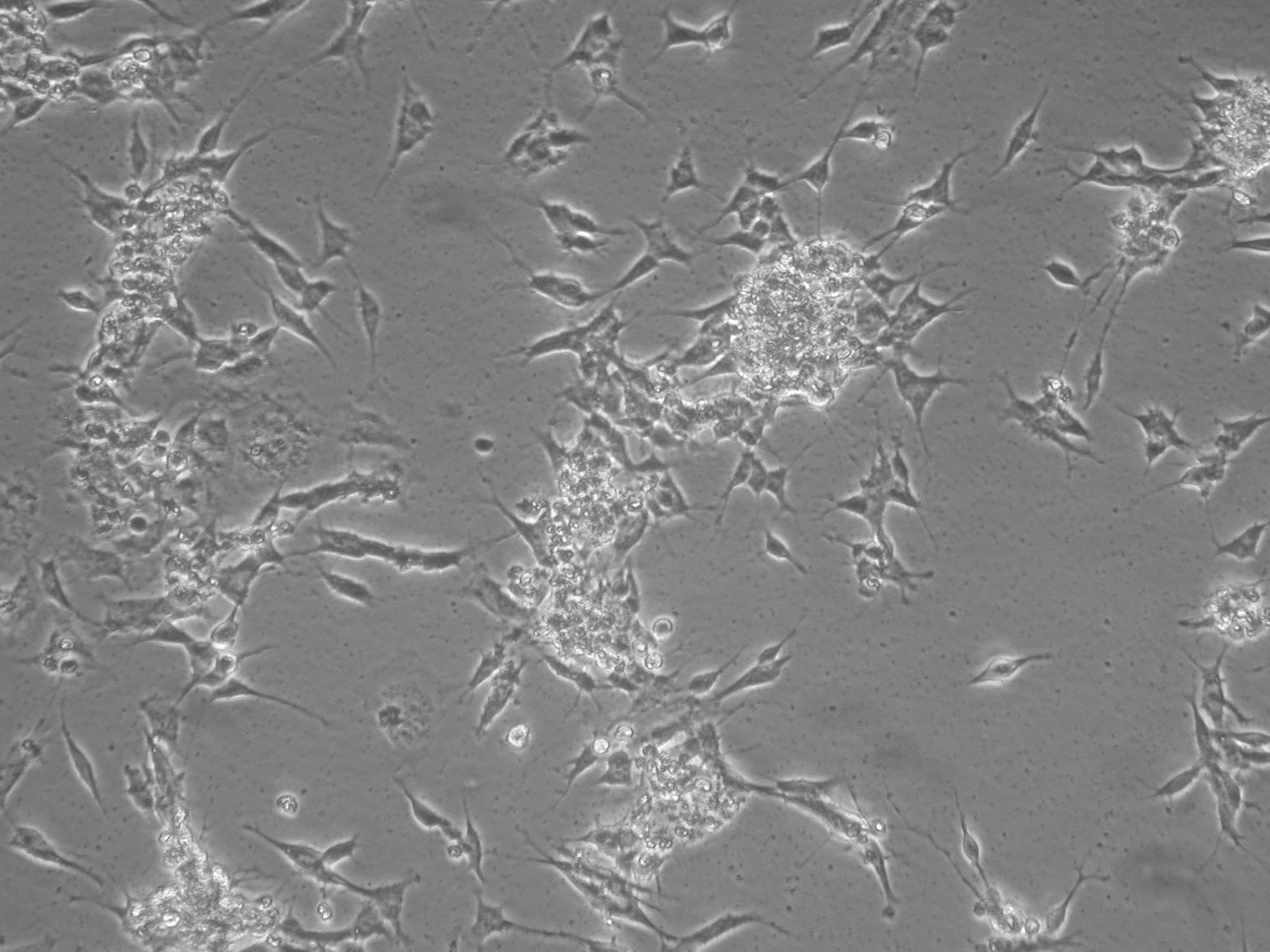

Supplement: S1 File — (ZIP) [file pone.0159082.s001.zip › S1 File/Fig 1A_SH-SY5Y SsnB 10 μM.jpg]

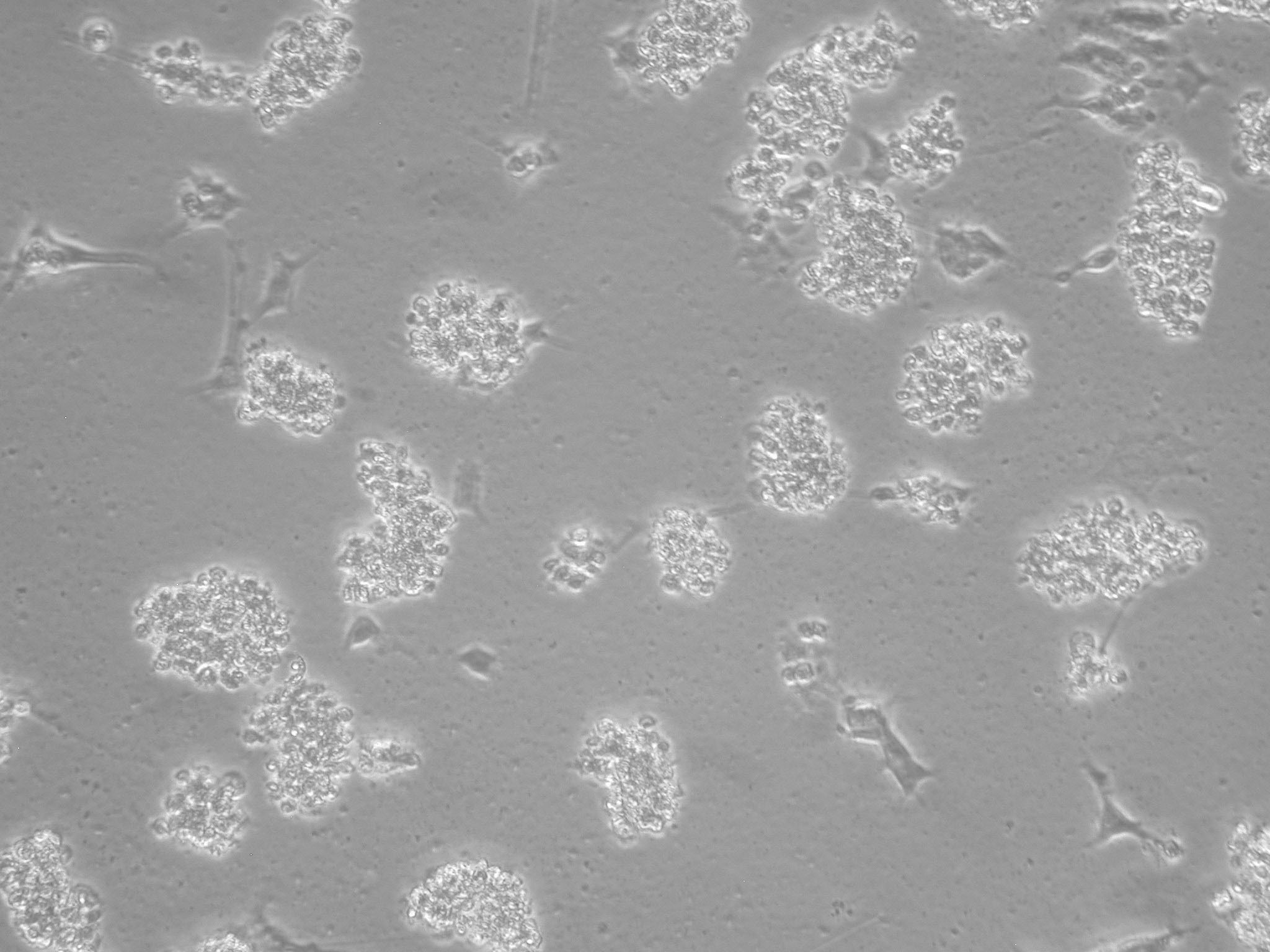

Supplement: S1 File — (ZIP) [file pone.0159082.s001.zip › S1 File/Fig 1A_SH-SY5Y SsnB 20 μM.jpg]

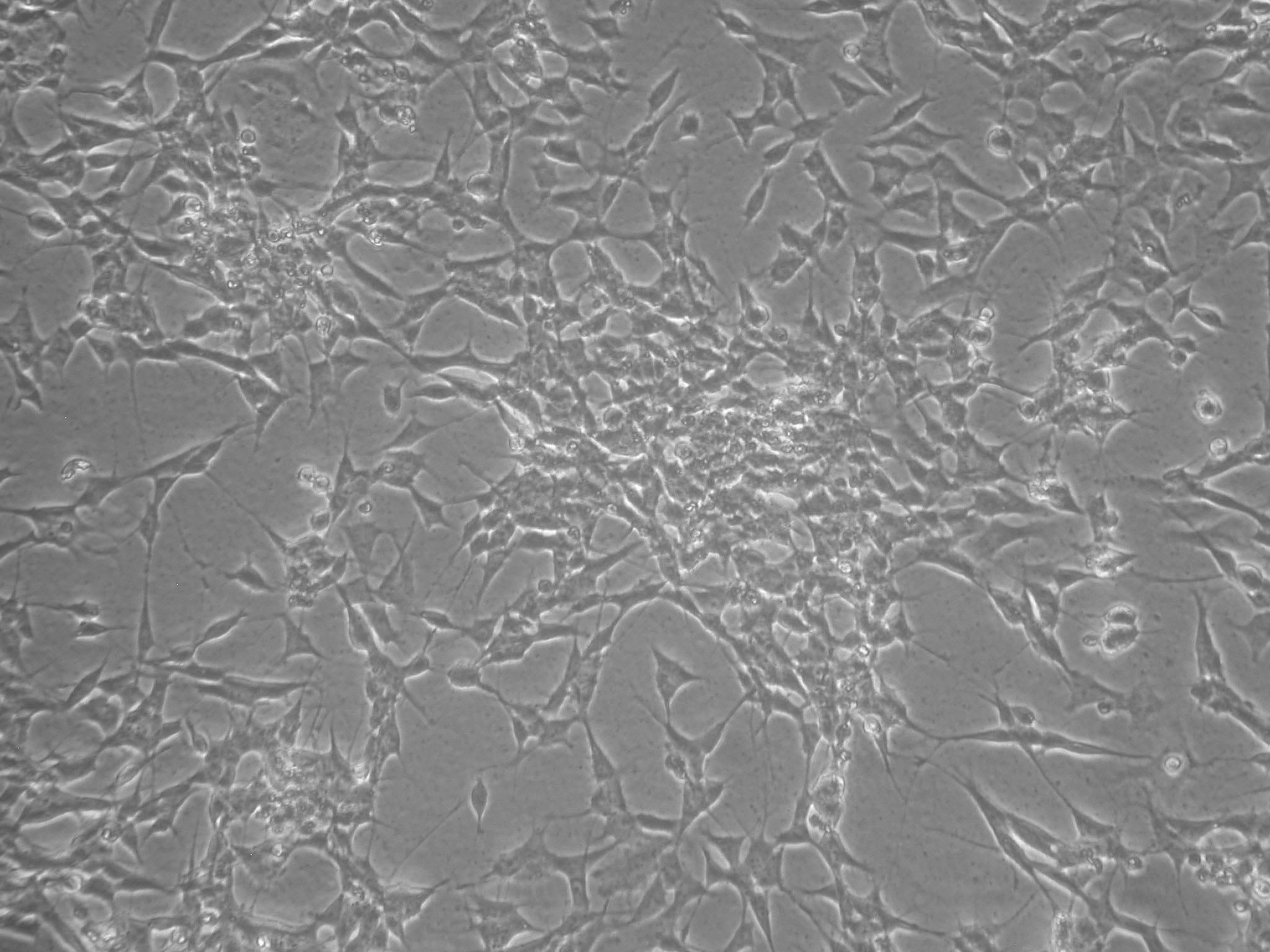

Supplement: S1 File — (ZIP) [file pone.0159082.s001.zip › S1 File/Fig 1A_SH-SY5Y SsnB 5 μM.jpg]

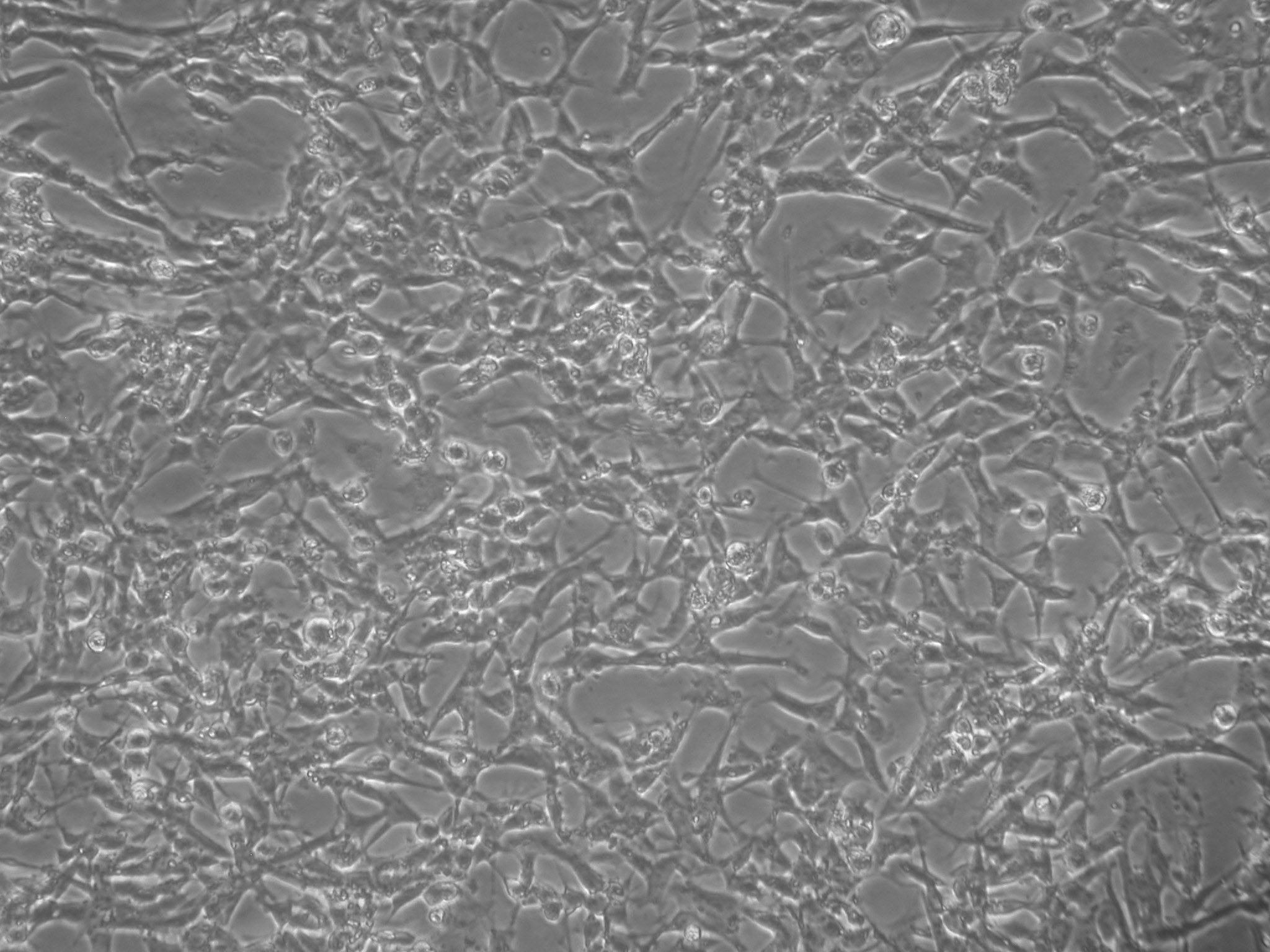

Supplement: S1 File — (ZIP) [file pone.0159082.s001.zip › S1 File/Fig 1A_SK-N-BE(2) DMSO.jpg]

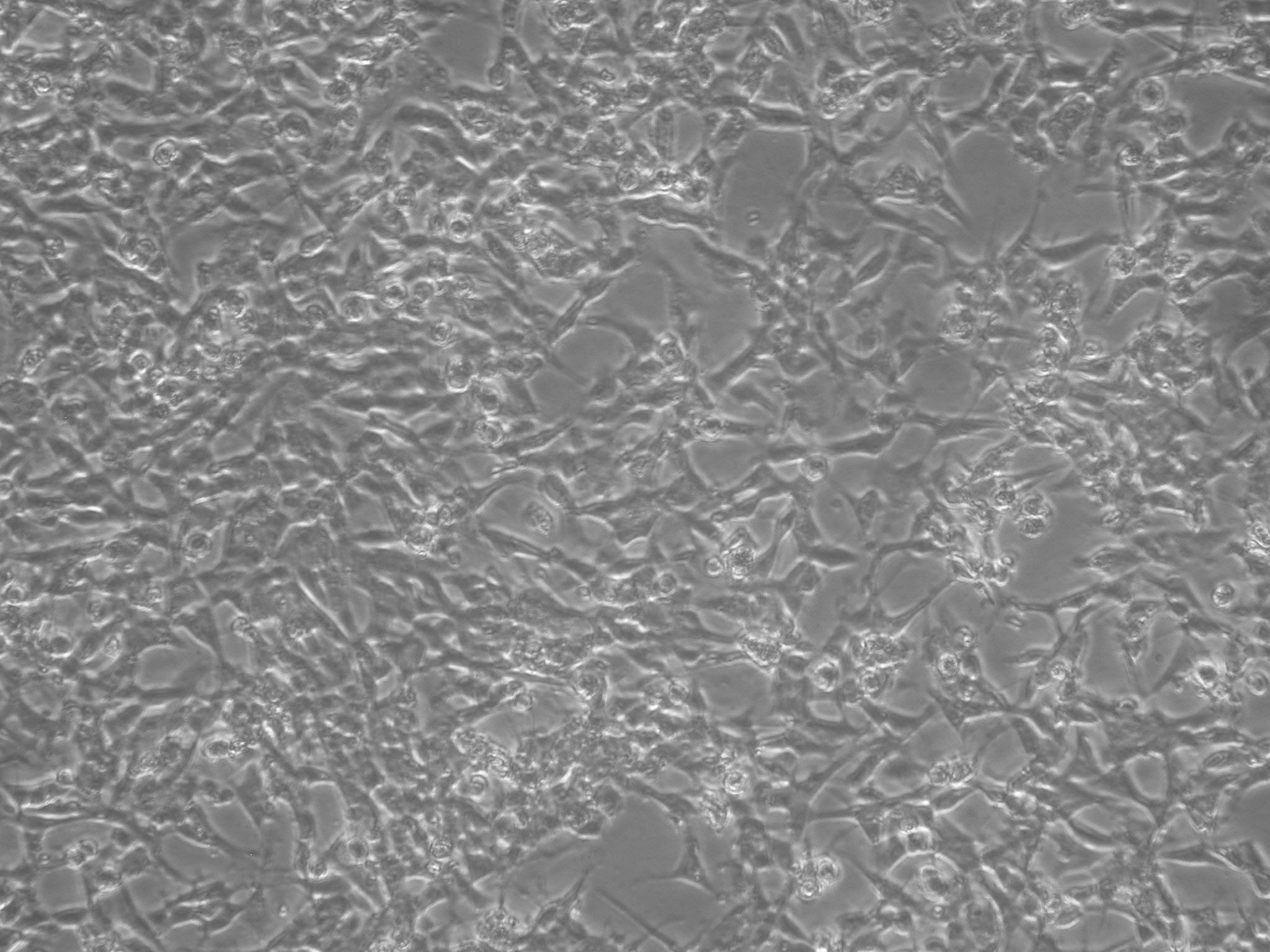

Supplement: S1 File — (ZIP) [file pone.0159082.s001.zip › S1 File/Fig 1A_SK-N-BE(2) SsnB 1 μM.jpg]

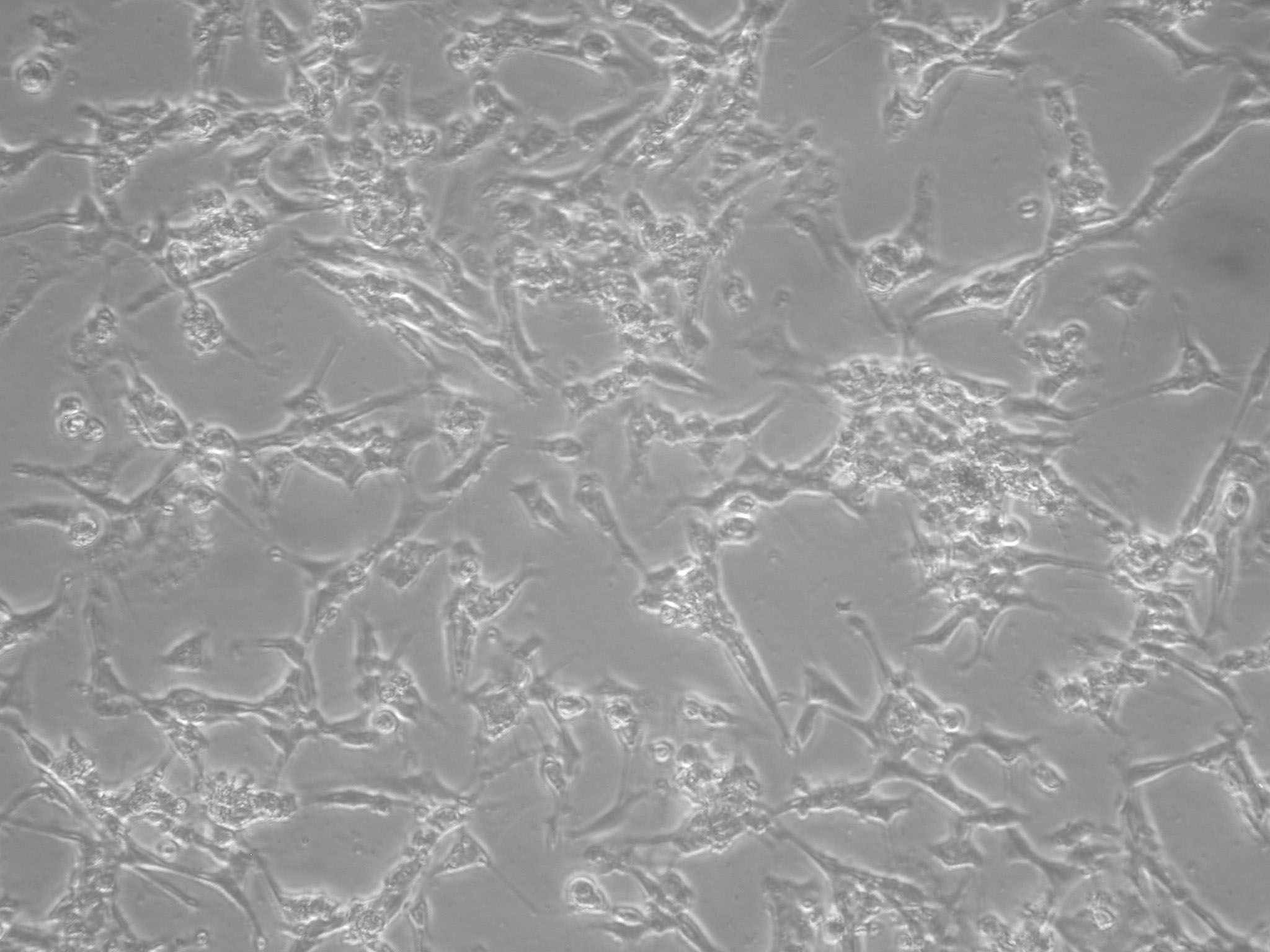

Supplement: S1 File — (ZIP) [file pone.0159082.s001.zip › S1 File/Fig 1A_SK-N-BE(2) SsnB 10 μM.jpg]

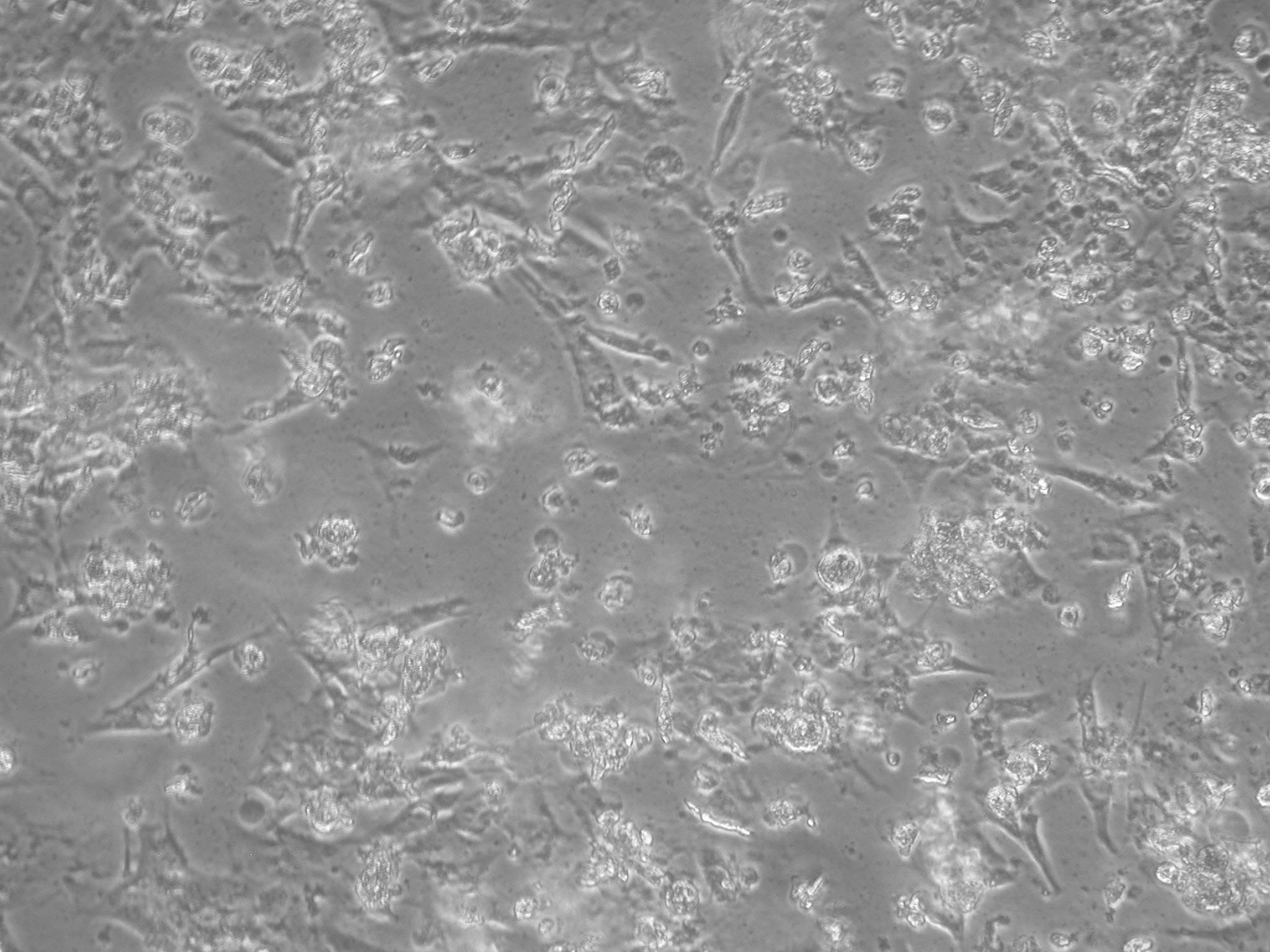

Supplement: S1 File — (ZIP) [file pone.0159082.s001.zip › S1 File/Fig 1A_SK-N-BE(2) SsnB 20 μM.jpg]

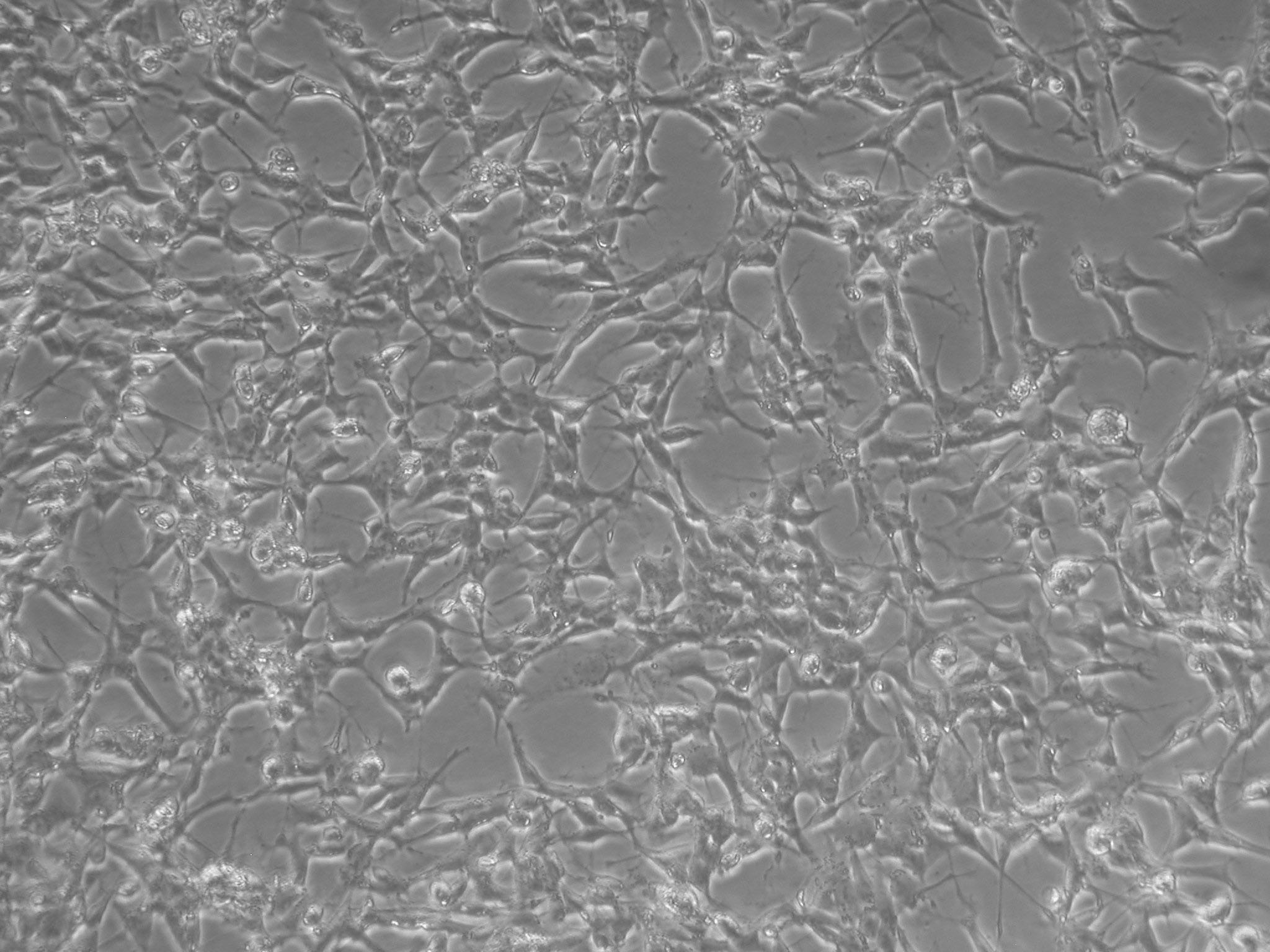

Supplement: S1 File — (ZIP) [file pone.0159082.s001.zip › S1 File/Fig 1A_SK-N-BE(2) SsnB 5 μM.jpg]

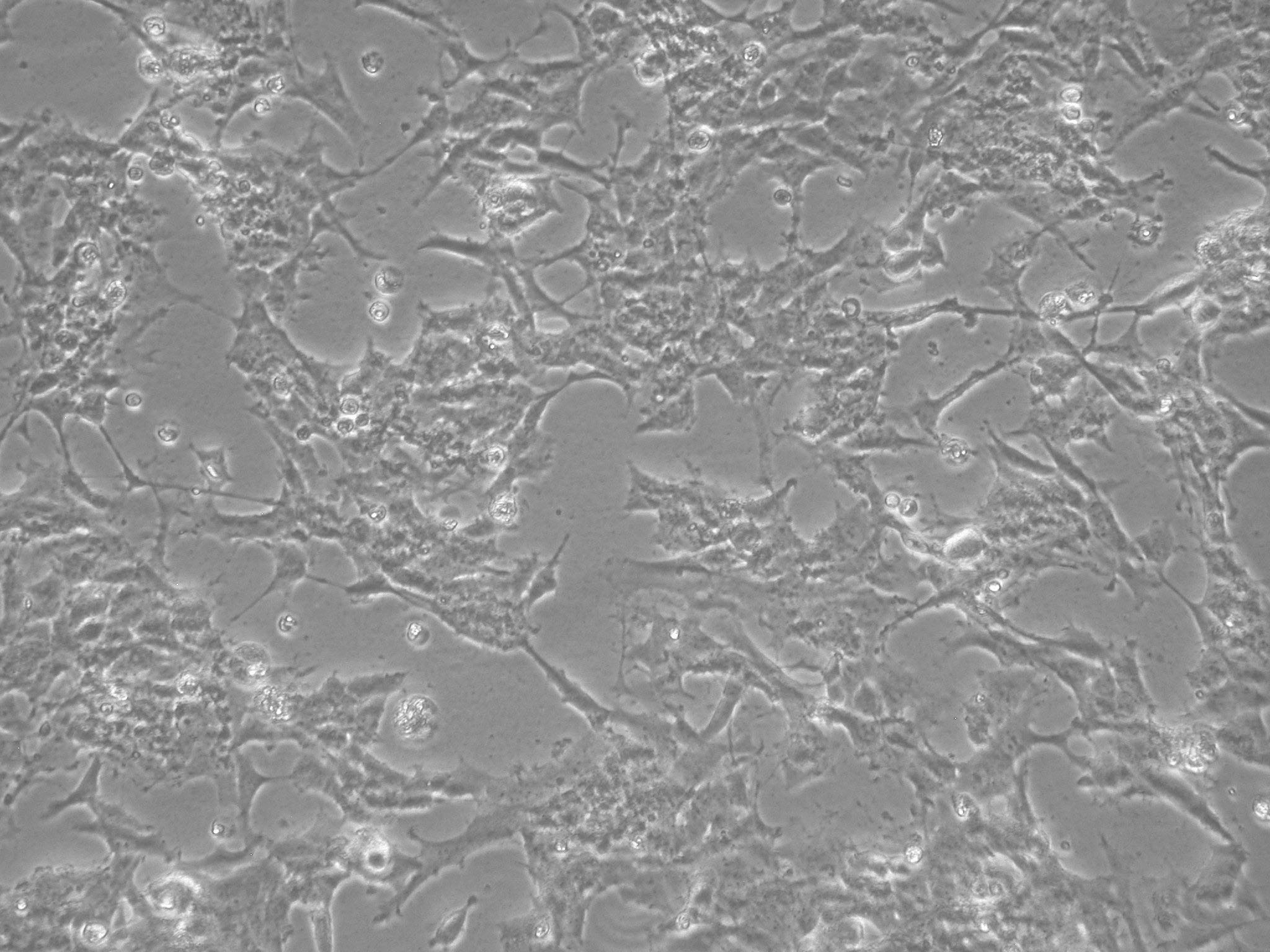

Supplement: S1 File — (ZIP) [file pone.0159082.s001.zip › S1 File/Fig 1A_SKNF-1 DMSO.jpg]

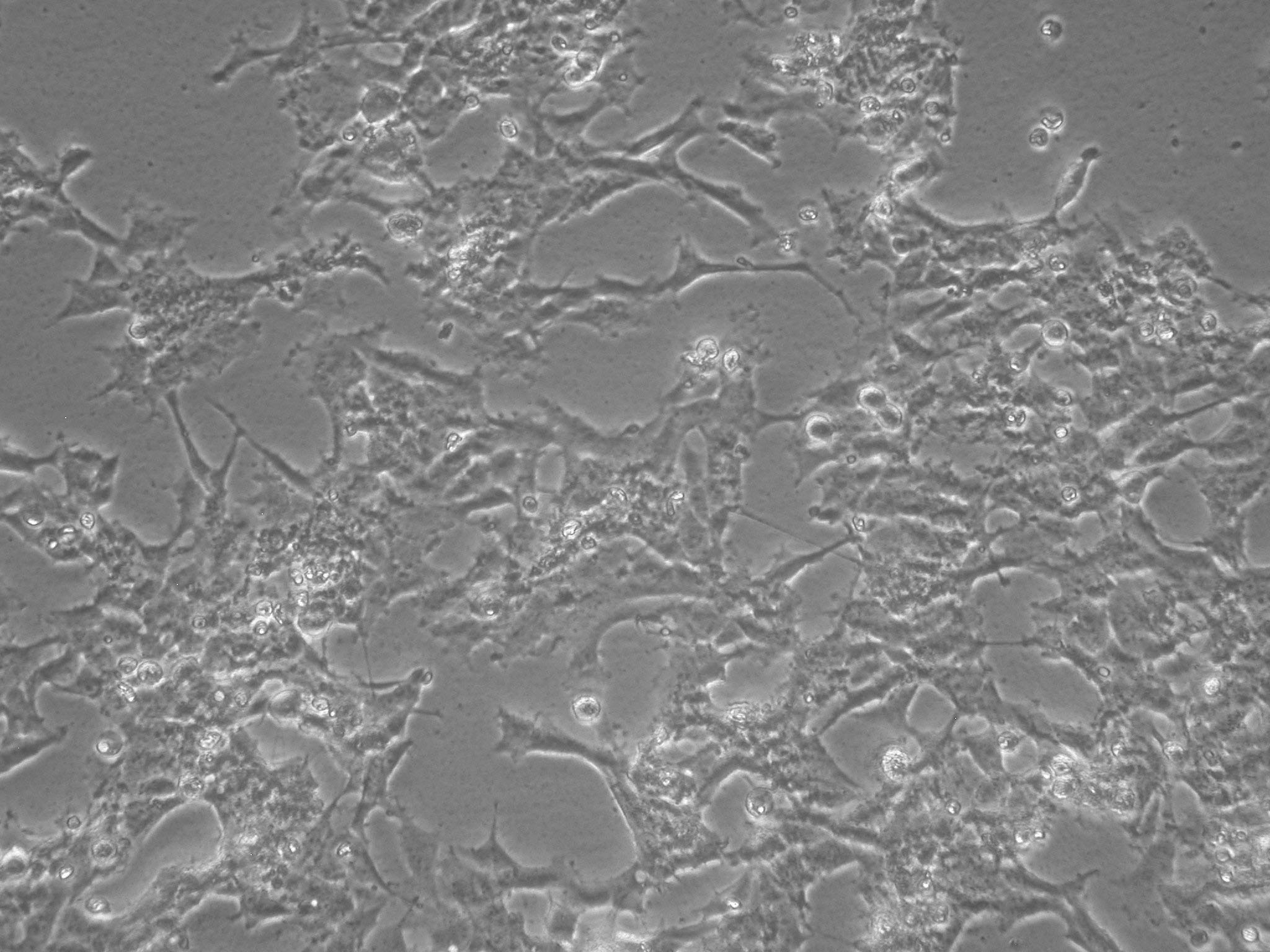

Supplement: S1 File — (ZIP) [file pone.0159082.s001.zip › S1 File/Fig 1A_SKNF-1 SsnB 1 μM.jpg]

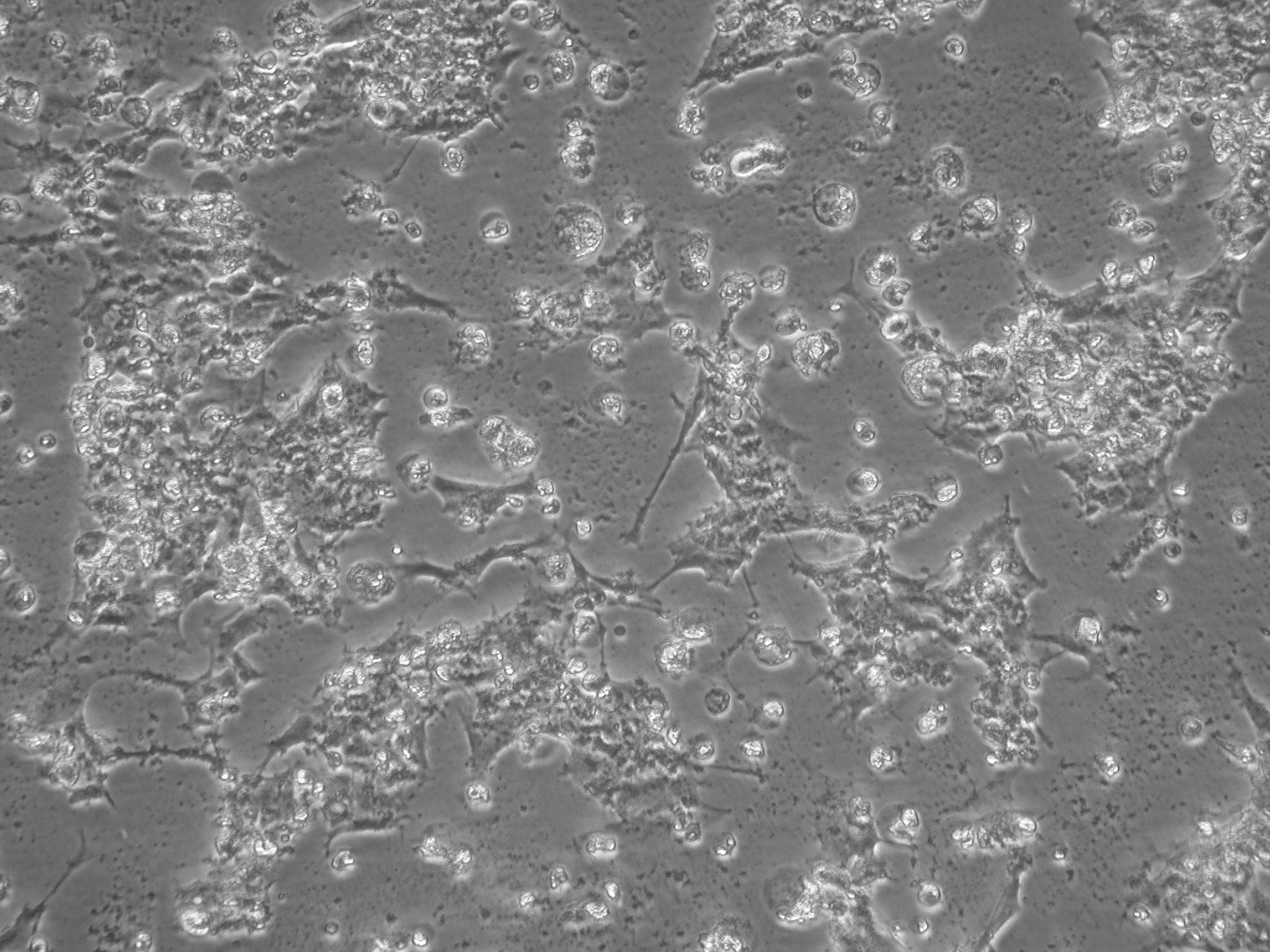

Supplement: S1 File — (ZIP) [file pone.0159082.s001.zip › S1 File/Fig 1A_SKNF-1 SsnB 10 μM.jpg]

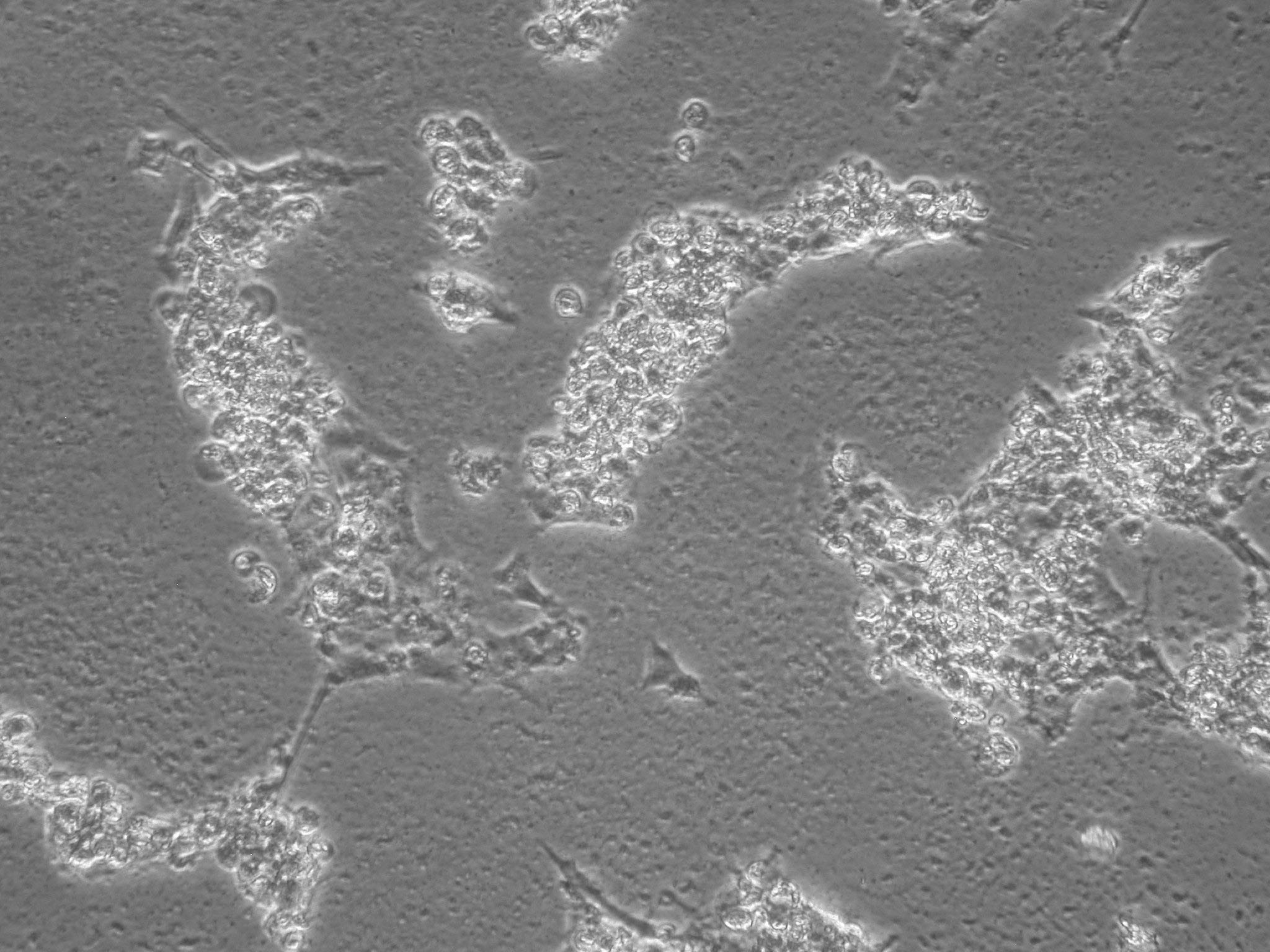

Supplement: S1 File — (ZIP) [file pone.0159082.s001.zip › S1 File/Fig 1A_SKNF-1 SsnB 20 μM.jpg]

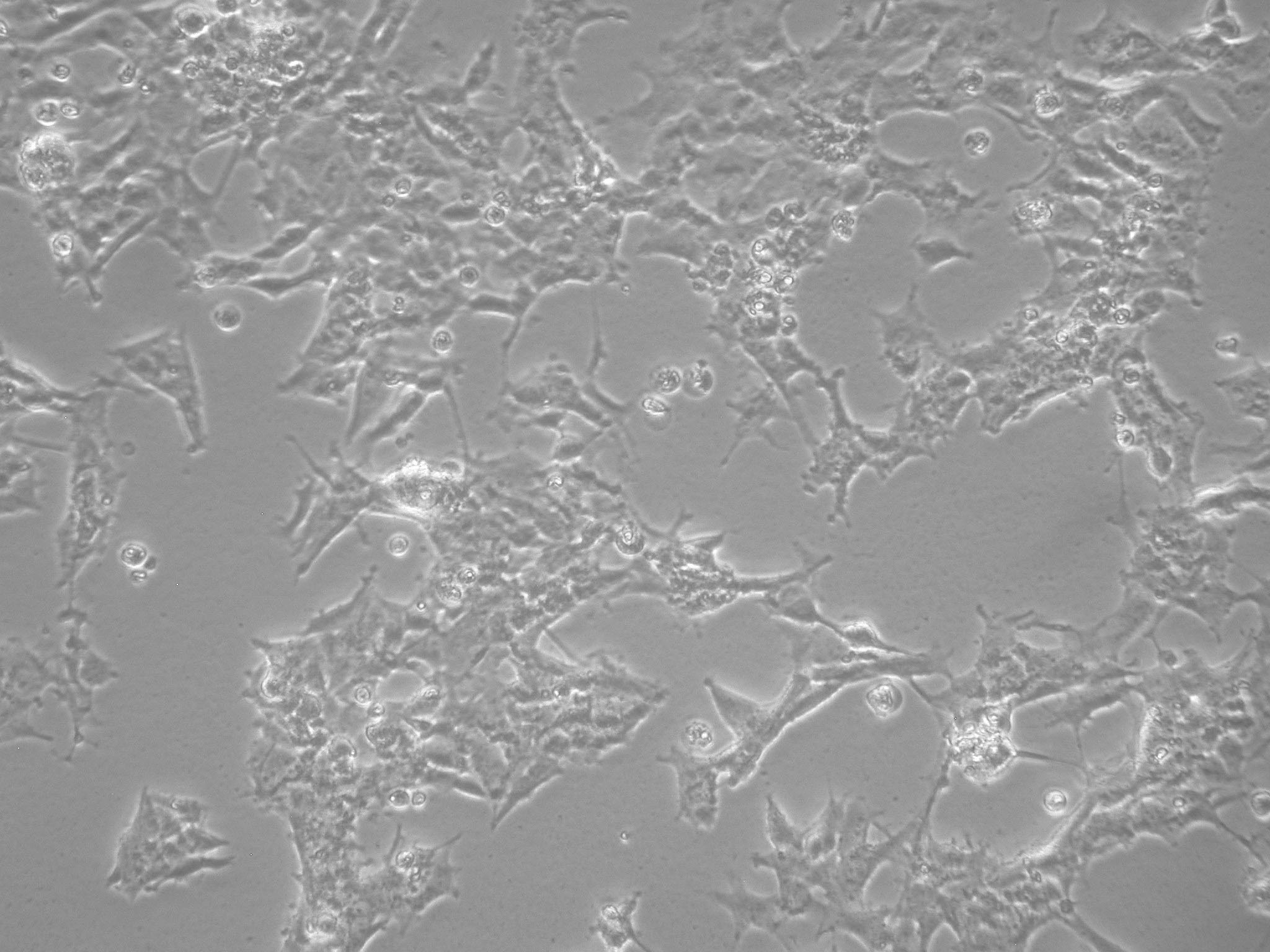

Supplement: S1 File — (ZIP) [file pone.0159082.s001.zip › S1 File/Fig 1A_SKNF-1 SsnB 5 μM.jpg]

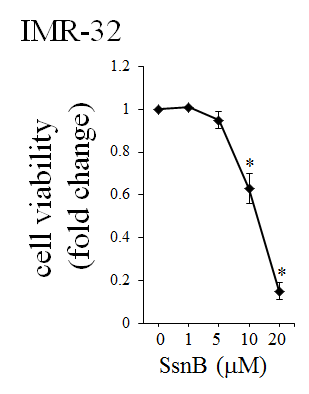

Supplement: S1 File — (ZIP) [file pone.0159082.s001.zip › S1 File/Fig 1B_IMR-32.tif]

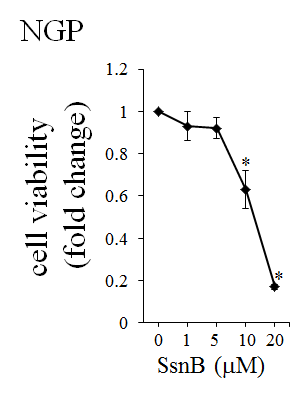

Supplement: S1 File — (ZIP) [file pone.0159082.s001.zip › S1 File/Fig 1B_NGP.tif]

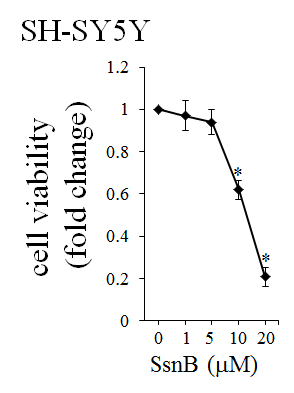

Supplement: S1 File — (ZIP) [file pone.0159082.s001.zip › S1 File/Fig 1B_SH-SY5Y.tif]

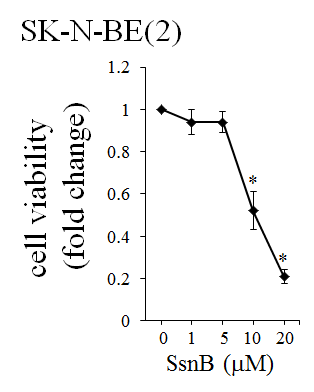

Supplement: S1 File — (ZIP) [file pone.0159082.s001.zip › S1 File/Fig 1B_SK-N-BE(2).tif]

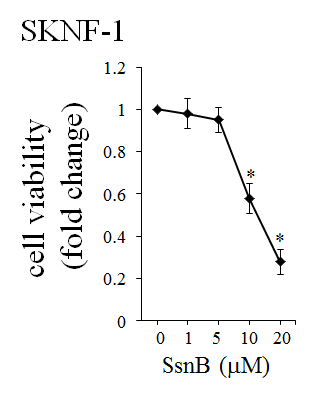

Supplement: S1 File — (ZIP) [file pone.0159082.s001.zip › S1 File/Fig 1B_SKNF-1.tif]

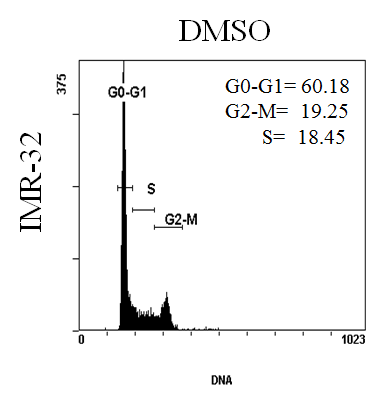

Supplement: S1 File — (ZIP) [file pone.0159082.s001.zip › S1 File/Fig 1C_IMR-32 DMSO.tif]

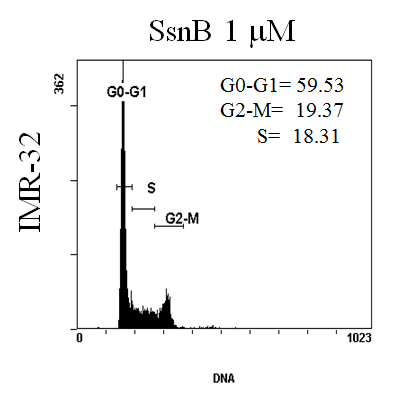

Supplement: S1 File — (ZIP) [file pone.0159082.s001.zip › S1 File/Fig 1C_IMR-32 SsnB 1 μM.tif]

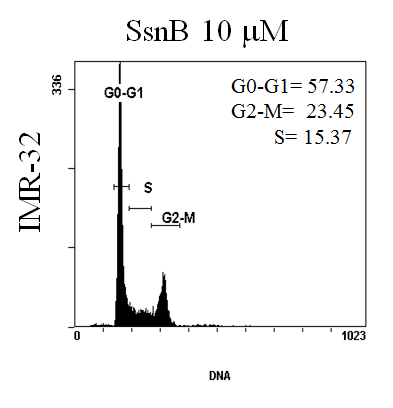

Supplement: S1 File — (ZIP) [file pone.0159082.s001.zip › S1 File/Fig 1C_IMR-32 SsnB 10 μM.tif]

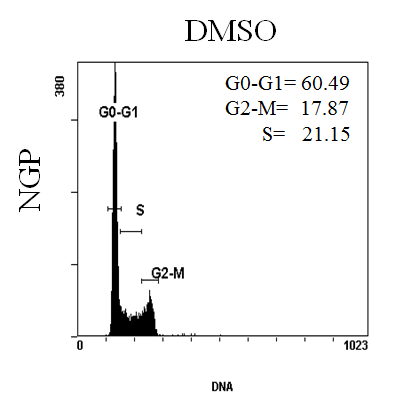

Supplement: S1 File — (ZIP) [file pone.0159082.s001.zip › S1 File/Fig 1C_NGP DMSO.tif]

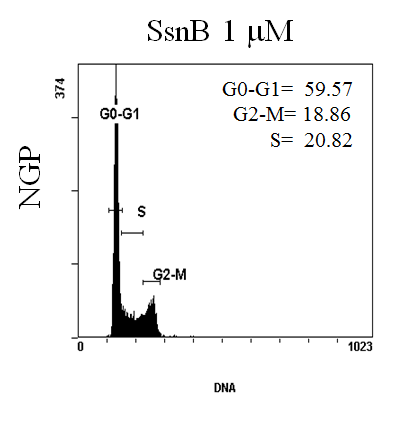

Supplement: S1 File — (ZIP) [file pone.0159082.s001.zip › S1 File/Fig 1C_NGP SsnB 1 μM.tif]

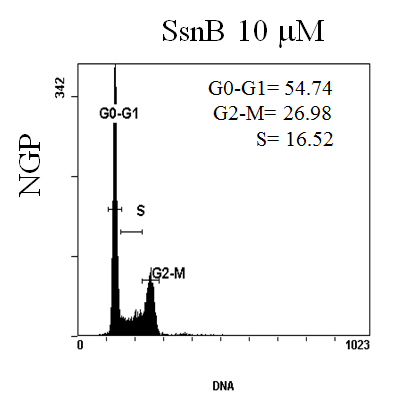

Supplement: S1 File — (ZIP) [file pone.0159082.s001.zip › S1 File/Fig 1C_NGP SsnB 10 μM.tif]

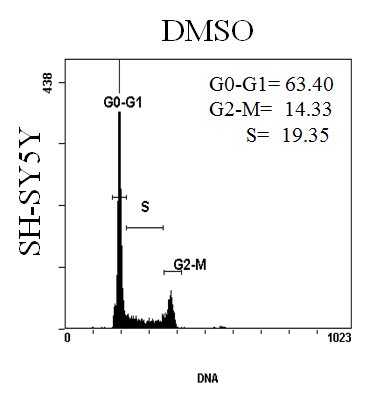

Supplement: S1 File — (ZIP) [file pone.0159082.s001.zip › S1 File/Fig 1C_SH-SY5Y DMSO.tif]

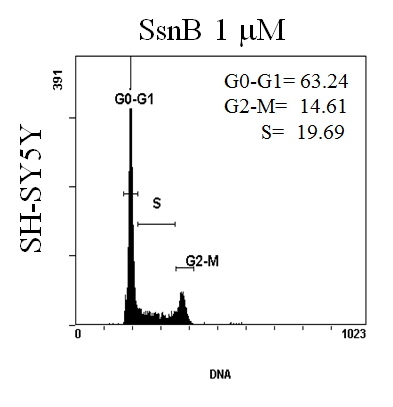

Supplement: S1 File — (ZIP) [file pone.0159082.s001.zip › S1 File/Fig 1C_SH-SY5Y SsnB 1 μM.tif]

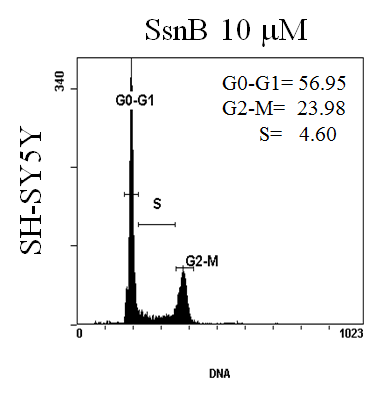

Supplement: S1 File — (ZIP) [file pone.0159082.s001.zip › S1 File/Fig 1C_SH-SY5Y SsnB 10 μM.tif]

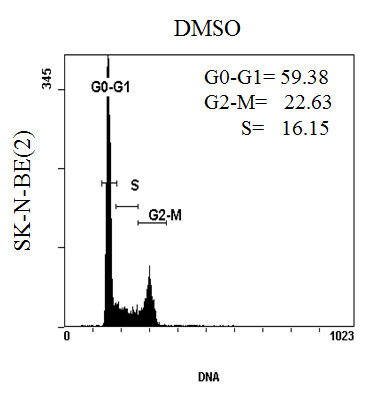

Supplement: S1 File — (ZIP) [file pone.0159082.s001.zip › S1 File/Fig 1C_SK-N-BE(2) DMSO.tif]

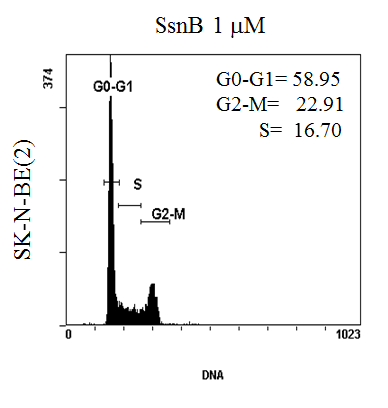

Supplement: S1 File — (ZIP) [file pone.0159082.s001.zip › S1 File/Fig 1C_SK-N-BE(2) SsnB 1 μM.tif]

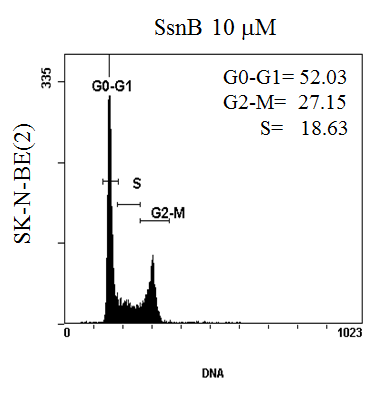

Supplement: S1 File — (ZIP) [file pone.0159082.s001.zip › S1 File/Fig 1C_SK-N-BE(2) SsnB 10 μM.tif]

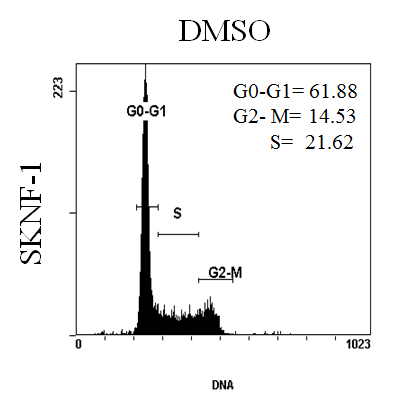

Supplement: S1 File — (ZIP) [file pone.0159082.s001.zip › S1 File/Fig 1C_SKNF-1 DMSO.tif]

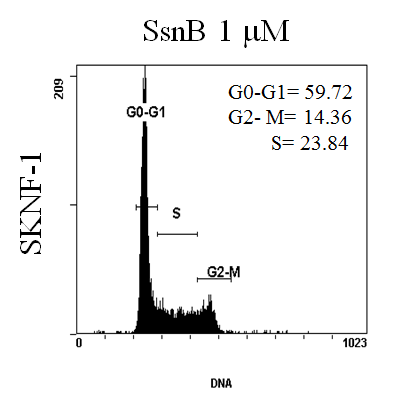

Supplement: S1 File — (ZIP) [file pone.0159082.s001.zip › S1 File/Fig 1C_SKNF-1 SsnB 1 μM.tif]

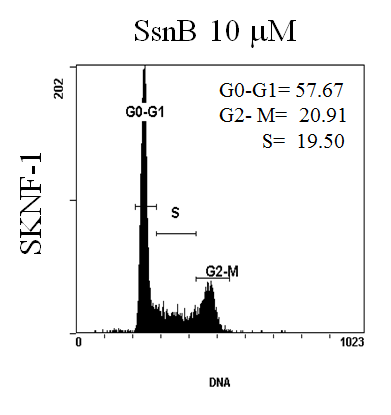

Supplement: S1 File — (ZIP) [file pone.0159082.s001.zip › S1 File/Fig 1C_SKNF-1 SsnB 10 μM.tif]
